# Digital Health Policy and Programs for Hospital Care in Vietnam: Scoping Review

**DOI:** 10.2196/32392

**Published:** 2022-02-09

**Authors:** Duc Minh Tran, C Louise Thwaites, Jennifer Ilo Van Nuil, Jacob McKnight, An Phuoc Luu, Chris Paton, Vietnam ICU Translational Applications Laboratory (VITAL)

**Affiliations:** 1 Oxford University Clinical Research Unit Ho Chi Minh City Vietnam; 2 Centre for Tropical Medicine and Global Health University of Oxford Oxford United Kingdom; 3 Nuffield Department of Medicine University of Oxford Oxford United Kingdom; 4 Department of Information Science University of Otago Otago New Zealand; 5 See Acknowledgments

**Keywords:** digital health, eHealth, policy, Vietnam, hospital care, data, health, electronic medical records, standards, compulsory, patient ID, administrative information, health insurance ID, mobile phone

## Abstract

**Background:**

There are a host of emergent technologies with the potential to improve hospital care in low- and middle-income countries such as Vietnam. Wearable monitors and artificial intelligence–based decision support systems could be integrated with hospital-based digital health systems such as electronic health records (EHRs) to provide higher level care at a relatively low cost. However, the appropriate and sustainable application of these innovations in low- and middle-income countries requires an understanding of the local government’s requirements and regulations such as technology specifications, cybersecurity, data-sharing protocols, and interoperability.

**Objective:**

This scoping review aims to explore the current state of digital health research and the policies that govern the adoption of digital health systems in Vietnamese hospitals.

**Methods:**

We conducted a scoping review using a modification of the PRISMA-ScR (Preferred Reporting Items for Systematic Reviews and Meta-Analyses Extension for Scoping Reviews) guidelines. PubMed and Web of Science were searched for academic publications, and *Thư Viện Pháp Luật*, a proprietary database of Vietnamese government documents, and the Vietnam Electronic Health Administration website were searched for government documents. Google Scholar and Google Search were used for snowballing searches. The sources were assessed against predefined eligibility criteria through title, abstract, and full-text screening. Relevant information from the included sources was charted and summarized. The review process was primarily undertaken by one researcher and reviewed by another researcher during each step.

**Results:**

In total, 11 academic publications and 20 government documents were included in this review. Among the academic studies, 5 reported engineering solutions for information systems in hospitals, 2 assessed readiness for EHR implementation, 1 tested physicians’ performance before and after using clinical decision support software, 1 reported a national laboratory information management system, and 2 reviewed the health system’s capability to implement eHealth and artificial intelligence. Of the 20 government documents, 19 were promulgated from 2013 to 2020. These regulations and guidance cover a wide range of digital health domains, including hospital information management systems, general and interoperability standards, cybersecurity in health organizations, conditions for the provision of health information technology (HIT), electronic health insurance claims, laboratory information systems, HIT maturity, digital health strategies, electronic medical records, EHRs, and eHealth architectural frameworks.

**Conclusions:**

Research about hospital-based digital health systems in Vietnam is very limited, particularly implementation studies. Government regulations and guidance for HIT in health care organizations have been released with increasing frequency since 2013, targeting a variety of information systems such as electronic medical records, EHRs, and laboratory information systems. In general, these policies were focused on the basic specifications and standards that digital health systems need to meet. More research is needed in the future to guide the implementation of digital health care systems in the Vietnam hospital setting.

## Introduction

Digital health systems such as electronic health records (EHRs) and patient administration systems used in hospitals in high-income countries (HICs) have been adopted with the dual aim of increasing the quality of patient care and improving hospital finances through cost reductions and new revenue streams. These systems are commonly introduced in response to major government initiatives, often with significant public funding [1]. Despite major challenges and high-profile failures [2], HICs have now reached the point where secondary use of data from digital health systems can, in some cases, enable hospitals and health care systems to become *learning health systems*, using routinely collected data to facilitate research and quality improvement [3]. In recent years, data from hospital-based digital health systems have been used to develop and implement innovative artificial intelligence (AI) systems for monitoring patients and providing clinical decision support (CDS) to health care providers [4].

Although the adoption of digital health systems in low- and middle-income countries (LMICs) such as Vietnam has largely only taken place within the last decade, these solutions have the potential to support the development of universal health coverage and projects working toward addressing sustainable development goals [5,6]. However, in resource-constrained settings, new health care information technologies are often implemented with insufficient funding, infrastructure, regulations, and computer literacy of the staff who will be using them [7-9]. These challenges may be able to be mitigated through the use of open-source software [8,10], mobile technologies [11], and cloud-based data infrastructure [12]. The adoption of new policies and standards (such as Health Level 7’s [HL7] Fast Healthcare Interoperability Resources [13]) may also enable simpler and more effective methods for health information exchange than was available to HICs in previous years [14].

Recent initiatives in Vietnam and other LMICs have sought to exploit the potential of digital technologies such as machine learning and low-cost wearable devices in improving critical care at an affordable cost [15,16]. For these innovations to attain scalability and sustainability, their research and development needs to consider the technical infrastructure and the regulatory frameworks that govern local technology adoption [5,6,15,16]. The Vietnamese government has recognized the role that digital health technologies can play in improving health care and optimizing administration processes [17], and along with building national health databases such as the national EHR system, new digital health policies and guidance have been promulgated by the Vietnam Ministry of Health (MoH) [18,19]. Awareness of the government’s current regulations and future directions for digital health and the local research evidence in this area is important for the introduction of these emergent technologies in Vietnamese hospitals. This scoping review aims to map and summarize the academic literature and government policies in the field of hospital-based digital health systems in Vietnam. We have chosen a scoping review methodology as it allowed us to effectively explore and summarize information from a wide range of sources, given the rapidly evolving nature of digital health in Vietnam [20,21].

## Methods

We conducted this review using a modification of the *PRISMA-ScR* (*Preferred Reporting Items for Systematic Reviews and Meta-Analyses Extension for Scoping Reviews): Checklist and Explanation* guidelines [22].

### Eligibility Criteria

The eligibility criteria are listed in Textbox 1.

**Eligibility criteria.**

**Inclusion criteria**

- Policies and guidance documents from the government that regulate and guide the adoption of digital health systems in Vietnam’s public hospitals. Academic publications that address digital health systems in Vietnam’s hospitals
- Policies and guidance documents that are functioning or to be mandated
- Documents written in Vietnamese or English

**Exclusion criteria**

- Policies and guidelines that have been replaced by a newer version
- Policies and studies that only examine technical aspects of the digital solution without discussing its adoption and implementation in clinical settings
- Stand-alone apps or digital solutions that are implemented in the hospital settings but not linked with a particular hospital-based information system such as hospital information management systems and electronic medical records

### Information Sources

We conducted searches on PubMed, Web of Science, and Google Scholar to identify academic literature. A structured search string (Multimedia Appendix 1) was built for the search on PubMed and Web of Science, and additional relevant publications identified during the full-text screening were retrieved and screened using Google Scholar.

A search of Vietnamese policies was conducted on the Thư Viện Pháp Luật database [23]. A total of 10 search queries were built and executed separately (Multimedia Appendix 1). All document titles resulting from each of the search queries were extracted and compiled in a Microsoft Excel spreadsheet, from which all duplicates were removed. The MoH Electronic Health Administration’s website was used to search for relevant documents that had not been found in the structured database search. Follow-up visits to the website were done throughout the study and at the end of the data-charting phase to identify newly released documents. Relevant documents that were referred to in the reviewed text but not found by the 2 former search strategies were searched for using Google.

No restriction on publication type and publication year was set.

### Data-Charting Process

A data-charting form was developed based on the research objectives and guidelines from the PRISMA-ScR guidelines. A draft version of the data-charting form was piloted during the full-text screening, from which discussions were made to modify and finalize the charting form.

All the charting tasks were conducted by one researcher (DMT) using a Microsoft Excel spreadsheet. The charted data were then reviewed by another researcher (CP), and follow-up discussions were arranged to resolve any disagreements between the 2 investigators.

For academic literature, the charting form collected information about the publication year, methods, study population, or source of information reviewed (if the publication is a review), the study scope, the digital health domain being studied, the context that the domain was investigating, and key findings. The charting form of government documents sought to extract information about the domain of intervention, policy enactment time, the intended purpose of the policy, and the ministry that mandated the document.

### Selection of Sources of Evidence

Academic publications were assessed through 3 steps including title, abstract, and full-text screening. Studies that did not meet the eligibility criteria in each step were ruled out from the screening list. Government policies and guidance were selected using a 2-stage process. In the first screening round, the title, introduction, and *scope and regulated entities* section of each document was screened to determine their eligibility. For documents that passed the first screening round, full text was screened in the second round. The selection was based on the eligibility criteria. Any uncertainty during the selection process was discussed between the authors for a final agreement.

## Results

### Overview

**Figure 1:**
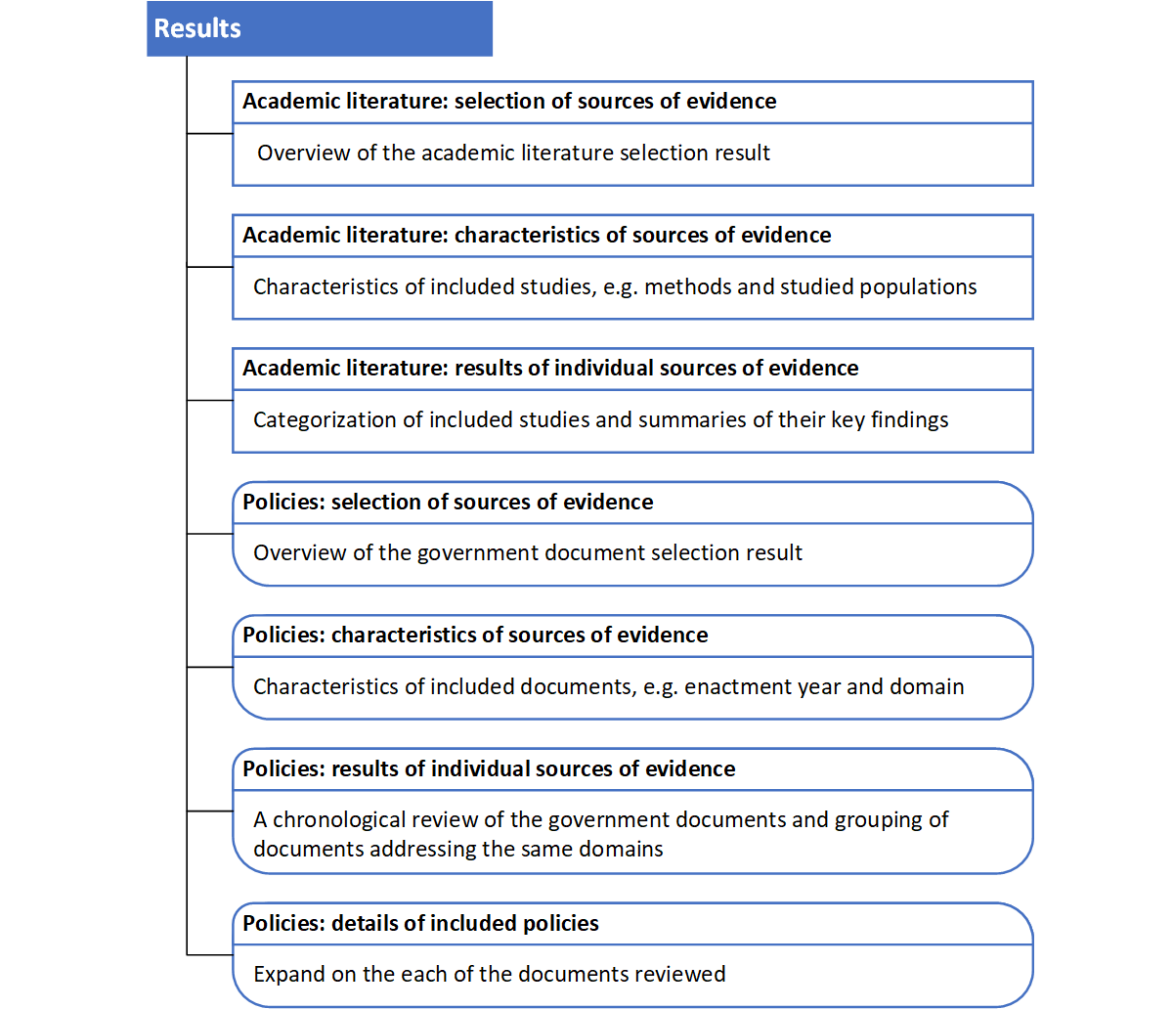
Layout of the *Results* section. The branches represent the subsections in the *Results* section and the information included therein. Square boxes and round boxes denote the Academic Literature and Policies subsections, respectively.

From the initial search results of 2033 academic publications and 266 government documents, 11 academic studies and 20 government documents were included in the review. We have organized the *Results* section into 2 major parts: academic literature and policies. Each part features the source selection process, characteristics of selected sources, and the results of individually selected sources. Figure 1 depicts the layout of the *Results* section.

### Academic Literature

#### Selection of Sources of Evidence

**Figure 2:**
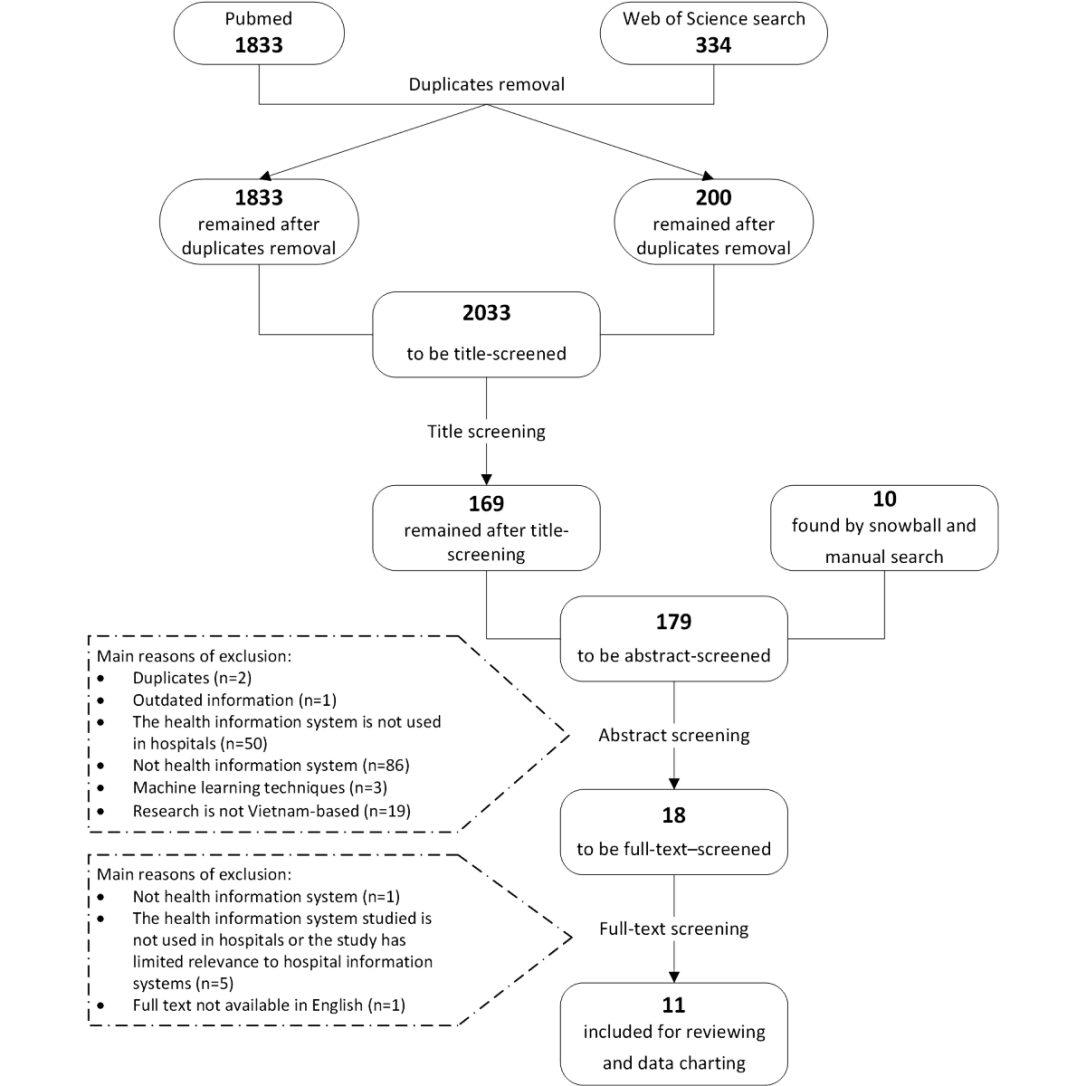
Summary of academic publication selection.

The PubMed and Web of Science searches of the academic literature returned 1833 and 334 articles, respectively. After the removal of duplicates, title screening was conducted on 2033 articles (1833 from PubMed and 200 from Web of Science), resulting in 169 marked *considered* for abstract screening. Titles were excluded if they did not imply any relation to the use of information technology (IT) in health care. In addition, the snowballing and manual search found an additional 10 studies, making it a total of 179 abstracts to be screened. The abstract screening ruled out 161 articles that did not meet the eligibility criteria and identified 18 articles eligible for the full-text review. The main reasons for exclusion during abstract screening were as follows: duplicated articles (n=2), the reviewed information was outdated to Vietnam’s current digital health situation (n=1), the interventions studied were neither of health information systems (n=86) nor of hospitals’ health information systems (n=50), the research was about machine learning techniques (n=3), and the research was not conducted in Vietnam (n=19). Of the full-texts screened, 11 articles were included in the final review. The full-text screening found 7 studies not eligible for the review, of which 1 did not examine health information systems, 5 studies investigated health information systems that were not implemented in hospitals or their findings were of limited relevance to the hospital information systems (HISs), and 1 study’s full text was not available in English (Figure 2).

#### Characteristics of Sources of Evidence

Characteristics of each study including title, authors, publication year, methods, studied population or source of information reviewed, and study scope are summarized in Table 1.

**Table 1:** Summary of included studies.

Study title	Reference	Methods	Studied population or source of information reviewed	Study scope
Design of Laboratory Information System for Health Care in Vietnam BK-LIS	Vu et al [24]	Case study	Common laboratory test results in Vietnam’s hospitals	Described how BK-LIS, a laboratory information system, was designed and developed to support the laboratory activities in Vietnam’s hospitals
A Design of Renal Dataflow Control and Patient Record Management System for Renal Department Environment in Vietnam	Vu et al [25]	Case study	Hemodialysis systems in Bach Mai Hospital and E Hospital, 2 central-level hospitals in Vietnam	A case study of the development of BK-HD manager, an IT^a^ solution that centrally manages the hemodialysis system in the hospital
Automatic Retrieving Data From Medical Equipment to Create Electronic Medical Records for an e-Hospital Model in Vietnam	Hai et al [26]	Case study	Electronic medical equipment commonly used in Vietnamese hospitals	Demonstrated technical solutions to automatically retrieve data from medical devices. The types of data include images and video data, laboratory test result data, and waveform data
Toward VNUMED for health care research activities in Vietnam	Vo et al [27]	Case study	EMRs^b^ from hospitals in Vietnam	Introduced VNUMED, an intermediate database that gathers data from EMRs to support health care research, and related challenges for its development in Vietnam
EMR Visualization for Patient Progress Tracking: Interface Design and System Implementation	VO et al [28]	Case study	Gastroenterologists and EMR data in Thong Nhat central-level hospital	Described the development and testing of EMR visualization, a visualization tool for patient progress tracking, using data from the hospital’s EMR system
Strategic Challenges Facing User- and Patient-Centered e-Health in Vietnam	Nguyen et al [29]	Review	IT use in health care in Vietnam before 2012	A review of the IT applications in Vietnam’s health sector before 2012. Challenges in implementing patient-centered eHealth in Vietnam were discussed
English-Based Pediatric Emergency Medicine Software Improves Physician Test Performance on Common Pediatric Emergencies: A Multicenter Study in Vietnam	Lin et al [30]	Multicenter, prospective, pretest–posttest study	203 physicians from 11 hospitals across Vietnam	PEMSoft^c^, a clinical decision support system, was tested against physicians’ performance on a multiple-choice exam
Toward an Electronic Health Record System in Vietnam: A Core Readiness Assessment	Hochwarter et al [31]	Document analysis, participant observation, and in-depth interview	Participant observation and document analysis was conducted in a department of a top-level hospital in Vietnam; in-depth interview with an MoH^d^ expert.	Investigation of the Vietnamese health system’s core readiness for electronic health record implementation
Open-Source LIMS^e^ in Vietnam: The Path Toward Sustainability and Host Country Ownership	Landgraf et al [32]	Reviewing the program reports	Reports from a national LIMS project using an open-source LIMS from 2008 to 2016	Described the building and scale-up of a national LIMS program for clinical and public health laboratories in Vietnam. Outcomes of the program and the lessons learned were discussed. A model for sustainability that could be applied to diverse laboratory programs was proposed
Electronic Health Record Readiness Assessment in Thái Binh Hospital, Vietnam	Nguyen [33]	Cross-sectional study using a scoring tool	Thái Binh provincial hospital	The study assessed the readiness for electronic health record implementation in Thái Binh hospital, Vietnam. The 4 main components of readiness included core readiness, technological readiness, learning readiness, and societal readiness
Artificial Intelligence vs Natural Stupidity: Evaluating AI^f^ Readiness for the Vietnamese Medical Information System	Vuong et al [34]	Nonsystematic review	The literature about AI in medicine worldwide, the literature about eHealth in Vietnam, and the Joint Annual Health Review of Vietnam from 2012 to 2016	An overview of AI research and applications in medicine worldwide, proposing a framework to evaluate AI readiness. The assessment of AI readiness in Vietnam’s health care sector using the proposed framework

^a^IT: information technology.

^b^EMR: electronic medical record.

^c^PEMSoft: Pediatric Emergency Medicine Software.

^d^MoH: Ministry of Health.

^e^LIMS: laboratory information management system.

^f^AI: artificial intelligence.

#### Results of Individual Sources of Evidence

The digital health domain that each study addressed, the context in which the domain was considered, year of data collection or reviewed evidence, and key findings of each study are presented in Table 2.

Among the 11 publications reviewed, 5 reported the development and testing of engineering solutions to gather, manage, or visualize data from the medical devices or information systems that were being used in the chosen hospitals. The 3 studies published in 2010 and 2011 described solutions to retrieve and manage data from laboratory devices or hemodialysis systems, whereas the 2 studies published in 2019 involved electronic medical record (EMR)-based solutions.

The other 6 articles addressed aspects of digital health implementation in Vietnam’s health care system. Among these, no study examined a specific clinical information system in the hospital setting. EHR implementation was discussed in the 2 readiness assessment studies, in which one interviewed an MoH staff member, whereas the other study surveyed health care workers in a provincial hospital. These stakeholders recognized the benefits offered by EHRs and expressed positive attitudes toward EHR adoption. However, there was a lack of IT infrastructure, basic IT use among the hospital staff such as regular communication via email, and IT training capacity in place for EHR implementation. Lin et al [30] evaluated physicians’ performance on a multiple-choice exam with the support of a system (CDS system [CDSS]). The participants improved their exam performance when using the CDS software compared with when not using the CDS software. However, the software was only used for testing purposes and not implemented in the studied hospitals. The national laboratory information management system program published by Landgraf et al [32] was largely used in public health laboratories rather than integrated into a hospital-based system. The 2 reviews by Nguyen et al [29] and Vuong et al [34] provided an overview of how health care facilities in Vietnam had implemented IT applications. Specifically, barriers for the development of patient-centered eHealth and AI in medicine in Vietnam were summarized, including the lack of national health databases, unstandardized data collection, and paper-based workflows. Importantly, most of the data analyzed in these 6 studies were collected in 2016 or earlier. The CDS performance and the EHR readiness assessments were conducted in the period from 2010 to 2014. The paper by Landgraf et al [32] was based on program reports from 2008 to 2016, whereas the review by Nguyen et al [29] provided insights into Vietnam eHealth before 2012. Discussions in the review by Vuong [34] were significantly carried out upon the analysis of the MoH annual reports from 2012 to 2016.

**Table 2:** Summary of the digital health domains and findings from academic publications.

Study	Digital health domain	Context in which the intervention was considered	Year of data collection or reviewed evidence^a^	Summary of findings relevant to digital health systems in hospitals in Vietnam
Vu et al [24]	Laboratory information system	Common laboratory test results in hospitals in Vietnam	2010 or earlier^b^	Presented how a laboratory information system was designed to solve the paper-based laboratory result management.
Vu et al [25]	Hemodialysis management system	Hemodialysis systems in Vietnam’s hospitals	2010 or earlier^b^	A central management system for hemodialysis machines in 2 hospitals in Vietnam was designed and tested.
Hai et al [26]	Data acquisition from medical devices	Electronic medical equipment commonly used in Vietnamese hospitals	2011 or earlier^b^	An engineering solution to automatically retrieve data from medical devices such as ultrasound, ECG^c^, and laboratory devices for personal health records.
Vo et al [27]	A database of EMR^d^ data	EMRs from hospitals in Vietnam	After 2019	The lack of standardized EMR use among Vietnamese hospitals posed a challenge for data gathering and research. VNUMED is a database that aims to collect and standardize data from different EMR systems.
VO et al [28]	Data visualization for EMRs	Data in EMR from a Vietnamese hospital	Between 2015 and 2019^b^	A data visualization app to track patient progress based on data collected from the local EMR system was developed and tested by the gerontologists as the end users. The testing results showed positive feedback from the end users regarding usability. The app was being updated for large-scale testing.
Nguyen et al [29]	Readiness for patient-centered eHealth	Health care in Vietnam before 2012	Before 2012	Provided an overview of information technology applications in Vietnam’s health care system from its beginning until 2011.
Lin et al [30]	CDS^e^	Physicians in hospitals	2010 to 2011	The study provided evidence that CDS technologies can improve physicians’ medical knowledge in the context of Vietnamese hospitals.
Hocwarter et al [31]	EHR readiness	Hospitals in Vietnam	2013	Provided evidence on the core readiness to a national EHR including the following: Identification of needs for future changes that will be addressed by the EHR system. Challenges posed by the status quo that demanded an EHR system: the existing system of medical records; quality of the existing record-keeping practice; the numbering system for medical records; patient identification methods in medical records; use of daily admissions and discharge lists; medical record archiving after patient discharge; medical record preservation when in archive; practice of ICD-10^f^ and use of ICD-10 in reality. Planning for the new EHR project by the Ministry of Health. Integration of technology: Integration with the current services; plan to integrate the EHR system with the existing hospital information systems; use of health informatics standards; the use of defined interfaces or gateways in data exchange.
Landgraf et al [32]	Laboratory information system	Mainly district health centers and public health laboratories in provinces with a high prevalence of HIV	2008 to 2016	Described the development, deployment, and operation of a national LIMS^g^ project using an open-source LIMS. Proposed factors for the sustainability of a health information system in Vietnam: (1) selection of appropriate technology, (2) capacity building and knowledge transfer, (3) financial viability, (4) leadership and management, and (5) alignment with national health strategies.
Nguyen et al [33]	EHR readiness	A provincial hospital in Vietnam	2013 to 2014	Provided evidence on EHR readiness in a Vietnamese provincial hospital, including the following: Core readiness: needs for change, planning, suitability of infrastructure, and integration of new technology with the existing services in the hospital. Technological readiness: need for information technology adoption and information technology infrastructure’s capability to implement an EHR. Learning readiness: information technology training for hospital and implementation staff. Societal readiness: Electronic communication with other organizations, data exchange between organizations, and sociocultural elements between health workers and patients.
Vuong et al [34]	Readiness for AI^h^ in medicine	Health care of Vietnam	2012-2018 (mainly 2012-2016)	Readiness for AI in medicine in Vietnam was assessed based on 3 factors: financial support, technological, and sociopolitical. In general, although AI in medicine research and political commitment was somewhat promising, the technical factor was seen as weak and inadequate.

^a^The period when primary data are collected or reviewed sources are published.

^b^On the basis of published year and reported grant period.

^c^ECG: electrocardiogram.

^d^EMR: electronic medical record.

^e^CDS: clinical decision support.

^f^ICD-10: International Classification of Diseases, Tenth Revision.

^g^LIMS: laboratory information management system.

^h^AI: artificial intelligence.

### Policies

#### Selection of Sources of Evidence

**Figure 3:**
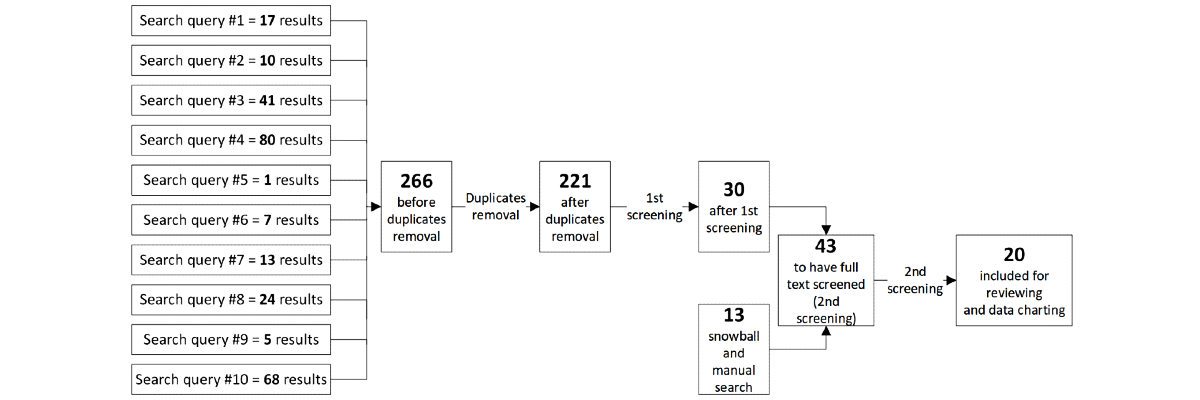
Summary of government document selection.

A total of 10 search queries on the *Thư Viện Pháp Luật* database returned 266 results. After removing duplicate titles, there were 221 results remaining in the first screening round. In total, 30 articles were selected from this screening phase with 191 articles excluded for the following reasons: unrelated to HIS adoption (n=157), limited relevance to HIS adoption (n=13), not a policy or guidance document (n=5), expired (n=13), and documents whose contents are covered in an included policy (n=3; Figure 3). Manual scanning on the MoH’s Electronic Health Administration website and snowballing from the reviewed documents helped identify 13 potential policies and guidance documents. Full text was screened for a total of 43 documents, which yielded 20 documents including for review and data charting.

#### Characteristics of Sources of Evidence

Table 3 presents an overview of the policies and guidance documents included. Characteristics of the documents were charted, including the enactment time points, policy ID numbers and titles, the digital health domains related to the policies, and the ministries that mandated the policies. Owing to the lengthy original titles, shorter denoted titles were created for easier reference of the documents in this paper.

**Table 3:** Summary of policy documents.

Valid from	Policy ID number and title	Denoted title	Domain	Ministry
December 2006	Decision 5573/QD-BYT year 2006 on Guideline for Hospital Information Management Systems [35]	The HIMS^a^ Guidance	Hospital information management system	MoH^b^
June 2013	Decision 2035/QD-BYT year 2013 on Terminology Systems and Data Exchange Standards Recommended for Health IT^c^ [36]	The Recommended Standards for HIT^d^	Standards for hospital information systems	MoH
October 2014	Decision 4159/QD-BYT year 2014 on Guidance on Ensuring Security of Electronic Health Data in the Health Sector [37]	The Cybersecurity Guidance	Cybersecurity in health organizations	MoH
March 2015 or January 2017^e^	Circular 53/2014/TT-BYT on Required Conditions for Provision of Health IT Activities [38]	The Required Conditions for HIT	Conditions for provision of health IT	MoH
October 2015	Decision 4495/QD-BYT year 2015 on Guideline for Developing Local Information Safety and Security Rules in Health Facilities [39]	The Guidance for Local Cybersecurity Policy	Cybersecurity in health organizations	MoH
October 2015	Decision 4494/QD-BYT year 2015 on Response Procedures for Information Safety and Security Issues in the Health Sector [40]	The Guidance for Cybersecurity Response	Cybersecurity in health organizations	MoH
September 2016	Decision 5004/QD-BYT year 2016 on The Architectural Framework of the Social Health Insurance Information System [41]	The Social Health Insurance EAF^f^	Electronic health insurance claim	MoH
June 2016	Decision 917/QD-BHXH year 2016 on Announcement of the Health Insurance Portal version 2 [42]	The Insurance Portal V2 Guidance	Electronic health insurance claim	Vietnam Social Security
September 2017	Decision 4210/QD-BYT year 2017 on Requirements for Standard and Format of Output Data Used in Management, Assessment and Reimbursement of Insurance-Paid Health Care Expenses [43]	The Claim Standardization Guidance	Electronic health insurance claim	MoH
August 2017	Decision 3725/QD-BYT year 2017 on Guidelines for Functionalities, Interoperability, Infrastructure and Human Resources for Establishing and Implementing Laboratory Information Systems at Healthcare Facilities [44]	The LIS^g^ Guidance	LIS	MoH
February 2018	Circular 54/2017/TT-BYT on Assessment Criteria for Information Technology Implementation in Healthcare Facilities [45]	The HIT Maturity Model	HIT maturity	MoH
July 2018	Circular 39/2017/TT-BTTTT on Technical Standards for IT Implementation in State Organizations [46] (replaced circular 22/2013/TT-BTTTT)	The Recommended Standards for IT in State Organizations	Standards for IT applications in state organizations	MIC^h^
March 2018	Circular 48/2017/TT-BYT on Regulations on Data Exchange in Management and Reimbursement of Health Insurance Claims [47]	The Electronic Claim Regulations	Electronic health insurance claim	MoH
December 2018	Decision 7603/QD-BYT year 2018 on The Service Coding System for Healthcare Management and Health Insurance Reimbursement version 6 [48]	The Terminology and Service Coding System version 6	Electronic health insurance claim	MoH
October 2019	Decision 4888/QD-BYT year 2019 on The Smart Health IT Implementation and Development Scheme from 2019 to 2025 [49]	The Smart HIT Scheme	Digital health strategies	MoH
March 2019	Circular 46/2018/TT-BYT on Regulations for Electronic Medical Records [50]	The Regulations for EMRs^i^	EMRs	MoH
November 2019	Decision 5349/QD-BYT year 2019 on Implementation Plan for Electronic Health Record [51]	The EHR^j^ Plan	EHRs	MoH
December 2019	Decision 6085/QD-BYT year 2019 on the eHealth Architectural Framework version 2.0 [52]	The EHAF^k^ version 2	Architectural framework	MoH
December 2020	Decision 5316/QD-BYT year 2020 on The Digital Transformation in Health Care Scheme Until 2025 and Navigated Toward 2030 [53]	The Digital Transformation Scheme	Digital health strategies	MoH
May 2020	Decision 2153/QD-BYT year 2020 on Regulations on Creation, Utilization and Management of Health ID [54]	The Health ID Regulation	National health ID	MoH

^a^HIMS: Hospital Information Management System.

^b^MoH: Ministry of Health.

^c^IT: information technology.

^d^HIT: health information technology.

^e^March 2015 or January 2017: applicable since March 2015 for organizations that had not implemented any health information systems and since January 2017 for organizations that had implemented health information systems.

^f^EAF: electronic architecture framework.

^g^LIS: laboratory information system.

^h^MIC: Ministry of Information and Communications.

^i^EMR: electronic medical record.

^j^EHR: electronic health record.

^k^EHAF: eHealth architectural framework.

#### Results of Individual Sources of Evidence

A total of 20 government documents were combined into 11 groups based on the digital health domains or subjects that they addressed. These groups included hospital information management systems, general and interoperability standards, cybersecurity in health organizations, conditions for the provision of health IT, electronic health insurance claims, laboratory information systems (LISs), health IT (HIT) maturity, digital health strategies, EMRs, EHRs, and eHealth architectural framework (EHAF). Table 4 presents these policy groups and the main purpose of each policy. In the following section, we have summarized the policies and their objectives in chronological order.

Most of the digital health policies reviewed were released and came into effect after 2013, excluding the Hospital Information Management System (HIMS) Guidance, which was enacted in 2006. The HIMS Guidance was an instruction regarding the functionalities and standards for HIMS used in state health facilities [35]. In 2013, the MoH promulgated guidance for interoperability standards and terminology systems applicable for health information systems [36]. Some of these are mandatory such as the HL7 messaging version 2 or 3, Digital Imaging and Communications in Medicine, SDMX-HD (Statistical Data and Metadata Exchange–Health Domain), and International Classification of Diseases, Tenth Revision (ICD-10) Clinical Modification, whereas the others are recommended, including the HL7 Clinical Document Architecture, HL7 Continuity of Care Document, World Health Organization Anatomical Therapeutic Chemical, and Logical Observation Identifiers Names and Codes. Standards for general IT applications are guided by the Ministry of Information and Communications (MIC) [46].

The 3 Cybersecurity Guidance documents for state health organizations were published in 2014 and 2015. The Cybersecurity Guidance covers 16 components of an IT system in a health organization such as network, databases, applications, account management, and data transmission [37]. As organizations using IT systems are required to develop and implement their own cybersecurity policy, the Guidance for Local Cybersecurity Policy provides general instructions for the making of these local policies [39]. Finally, the Guidance for Cybersecurity Response is a standard procedure to identify, classify, report, and handle cybersecurity issues in health facilities [40]. In March 2015, the Required Conditions for HIT announced the essential criteria that organizations must meet when implementing HIT in Vietnam [38]. Accordingly, organizations must satisfy specific requirements for IT infrastructure, information security, IT workforce, and implementation of several technologies.

In 2016 and 2017, the MoH and the Vietnam Social Security (VSS) initiated the electronic social health insurance claim system, which was accompanied by the promulgation of several regulations and guidance. First, the Social Health Insurance Electronic Architectural Framework was published, describing the system’s structure and operation [41]. A web-based portal was established, on which health providers can submit claims and check insurance information. To provide instructions for health facilities on using the portal, the VSS published the Insurance Portal V2 Guidance [42]. As claim data must be collected and formatted in a standardized manner, the Claim Standardization Guidance is intended to provide the necessary instructions for health facilities on claim preparation [43]. The Vietnam social health insurance system uses a national terminology and service coding system to form billing codes from medical documentation. In 2018, this coding system was in its sixth iteration [48]. Finally, the Electronic Claim Regulations defines the responsibilities of health providers and insurance agencies surrounding claim sharing and feedback [47].

**Table 4:** Main purposes of the policies.

Group of domains and denoted title			Purpose of the document		Valid from
**Hospital information management system**					
	The HIMS^a^ Guidance	A guidance for functionalities and standards of HIMSs		December 2006	
**General and interoperability standards**					
	The Recommended Standards for HIT^b^	A list of nomenclature systems and interoperability standards that the MoH^c^ required or recommended for health information systems		June 2013	
	The Recommended Standards for IT^d^ in State Organizations	A list of general IT standards that the MIC^e^ required or recommended for health information systems		July 2018	
**Cybersecurity in health organizations**					
	The Cybersecurity Guidance	A comprehensive guidance of cybersecurity measures for health facilities		October 2014	
	The Guidance for Local Cybersecurity Policy	A guideline for developing organization information safety and security policies for health facilities		October 2015	
	The Guidance for Cybersecurity Response	A guidance for classifying, identifying, reporting, and handling cybersecurity issues in health facilities		October 2015	
**Conditions for provision of health IT**					
	The Required Conditions for HIT	Criteria that health facilities must satisfy when implementing digital health systems. Four areas addressed in the circular include IT infrastructure, information security, human resource, and specific criteria for some HIT systems		March 2015 or January 2017	
**eHealth insurance claim**					
	The Social Health Insurance EAF^f^	Explaining the architecture model for the social health insurance information system to be built by the MoH		September 2016	
	The Insurance Portal V2 Guidance	Announcing the launching of the Health Insurance Portal version 2 with an installation manual attached		June 2016	
	The Claim Standardization Guidance	A guidance for standardization of claims data including variable definition and data standards		September 2017	
	The Electronic Claim Regulations	Responsibilities of the health care organizations and the insurance agencies in electronic claim exchange and investigation		March 2018	
	The Terminology and Service Coding System version 6	A common list of health services with the relevant codes used in the social health insurance claim and reimbursement, updated to version 6		December 2018	
**LIS^g^**					
	The LIS Guidance	A guidance for functionalities and standards of LISs		August 2017	
**HIT maturity**					
	The HIT Maturity Model	Seven levels of HIT application applicable for health care organizations, made of 8 key components and capabilities. Criteria for each HIT level and component were provided herein.		February 2018	
**Digital health strategies**					
	The Smart HIT Scheme	Presenting the agenda to develop and implement digital and smart technologies in Vietnam’s health care for the period from 2019 to 2025		October 2019	
	The Digital Transformation Scheme	Presenting the agenda to comprehensively implement IT in Vietnam’s health care until 2025 and navigated toward 2030		December 2020	
**EMRs^h^**					
	The Regulations for EMRs	Criteria for EMR development and implementation to abide by Health Care Law and replace paper medical records		March 2019	
**EHR^i^**					
	The EHR plan	The national plan of the MoH to build and implement the EHR system in Vietnam		November 2019	
	The Health ID Regulation	The health ID system used for eHealth data of Vietnamese residents		May 2020	
**Architectural framework**					
	The EHAF^j^ version 2	Explaining the architecture model of key IT systems and databases built by the MoH		December 19	

^a^HIMS: health information management system.

^b^HIT: health information technology.

^c^MoH: Ministry of Health.

^d^IT: information technology.

^e^MIC: Ministry of Information and Communications.

^f^EAF: electronic architectural framework.

^g^LIS: laboratory information system.

^h^EMR: electronic medical record.

^i^EHR: electronic health record.

^j^EHAF: eHealth architectural framework.

Guidance for LISs was published in August 2017, addressing the functionalities and standards required for such systems in Vietnam [44]. From 2018 to 2020, the MoH issued several policies that govern key digital health systems such as the maturity model for HIT, EMRs, and EHRs. From February 2018, the *HIT Maturity Model* began to be used as a roadmap for health care organizations to build and upgrade their digital health system [45]. Similar to the Healthcare Information and Management Systems Society’s Analytics Electronic Medical Record Adoption Model [55], this maturity model features 7 levels of HIT application, each of which is built on a specific set of criteria. The most basic level (level 1) only requires the implementation of HISs to manage outpatient services, whereas the highest level (level 7) defines a paperless environment that deploys a comprehensive range of HIT such as HIS, EMR, LIS, radiology information system and picture archiving and communication systems (RIS-PACS), and CDSS with interoperability between the systems. EMR adoption is governed by the *Regulations for EMRs*, enacted in March 2019 [50]. The circular sets out the requirements for EMRs so that their use abides by Health Care Law and is eligible to completely replace paper medical records. In the same year, the MoH announced a plan to roll out EHRs on a nationwide scale, starting in municipal cities [51]. Although EMRs serve inpatient care and can vary in their specifications depending on the specialty in which they are implemented, the EHR system can be seen as the digital version of the paper-based primary health record [56] and is a lifetime health document of every Vietnamese resident. Information in EHRs is mainly collected and updated by the district health facilities and outpatient clinics. The MoH has also announced the National Health ID system in the Health ID Regulation [54], in which each health ID number is unique and represents an individual for their lifetime.

**Figure 4:**
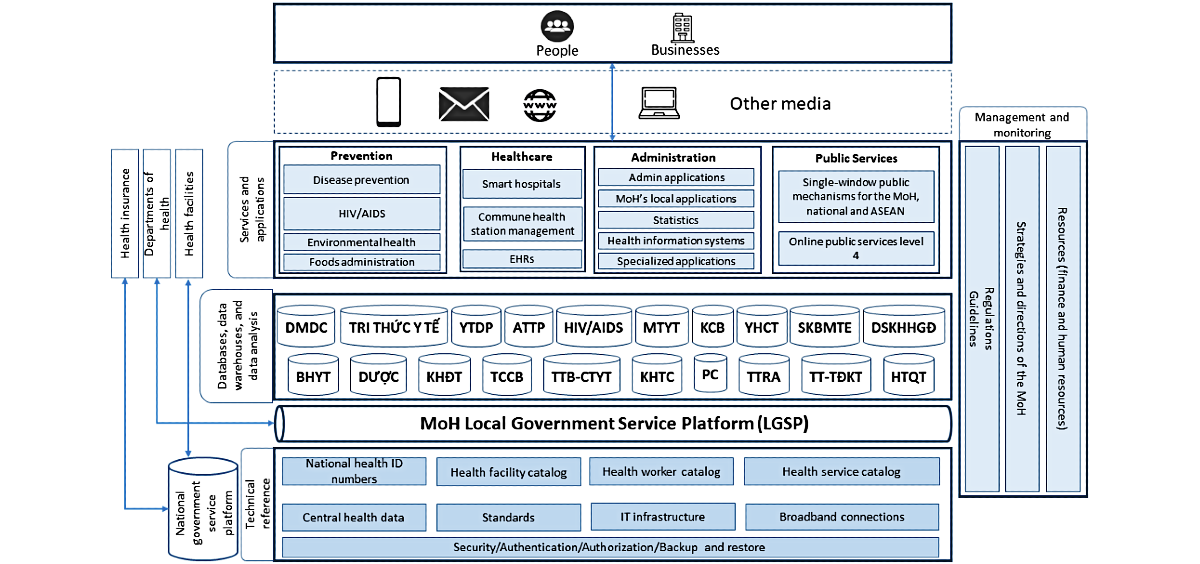
The Ministry of Health’s eHealth Architecture Framework version 2.0 (adapted from the Vietnam Ministry of Health [52]). The following are English translations for the abbreviations—ATTP: food safety; BHYT: health insurance; DMDC: reference catalogs; DSKHHGD: population planning; DƯỢC: pharmacy; HTQT: international collaboration; KCB: health care; KHĐT: research and training; KHTC: finance; MTYT: environmental health; PC: legislation; SKBMTE: maternal and child health; Tri thức y tế: health knowledge; TCCB: human resources; TTB-CTYT: medical devices and infrastructure; TTRA: inspection; TT-TĐKT: reward; YHCT: traditional medicine; YTDP: preventive medicine. EHR: electronic health record; IT: information technology; MoH: Ministry of Health.

An overall picture of the IT systems that the MoH has been developing is depicted in their EHAF, presented in Figure 4 (adapted from the Vietnam MoH [52]). The framework was first published in 2015 [57] and subsequently updated in 2018 [58] and 2019 with the EHAF version 2 [52]. According to the EHAF version 2, EMRs and EHRs are the 2 main components of the health care building block. In 2019, the MoH announced the Smart HIT Scheme, presenting an agenda to develop and implement digital and smart technologies in Vietnam’s health care for the period from 2019 to 2025 [49]. By the end of 2020, the Digital Transformation Scheme was published, setting the goals to implement IT in multiple areas of the health sector including state administration, cashless payment and telehealth, disease prevention and primary care, and health care [53].

#### Details of Included Policies

In the subsequent sections, we have expanded on the key policies in each digital health domain.

#### Architectural Framework: The EHAF Version 2

The framework is intended to guide the future developments of eHealth in the public sector, ensuring their compatibility with the current components and directed toward common goals.

The EHAF version 2.0 (decision 6085/QD-BYT year 2019 on the eHealth Architectural Framework Version 2.0 [52]) has 7 layers including the following:

1. End users (people and businesses)
2. Communication channels (means to communicate with applications and services of the MoH such as computers, smartphones, and information portals)
3. Services and applications layer. This layer comprises 4 building blocks including disease prevention, health care, administration, and public services
4. Databases and data analysis tools layer. This includes multiple databases specialized for each health area managed by the MoH, for example, traditional medicine, pharmacy, and health insurance
5. The MoH’s Local Government Service Platform. This platform provides shared supporting services for the upper layers. The MoH’s Local Government Service Platform is also able to communicate with other ministries and provinces through the National Government Service Platform
6. Technological infrastructure layer
7. Management and monitoring layer

An illustration of the EHAF version 2.0 can be found in Figure 4 (adapted from the Vietnam MoH [52]).

#### Digital Health Strategies

##### The Smart HIT Scheme

The Smart HIT Scheme (decision 4888/QD-BYT year 2019 on the Smart HIT Implementation and Development Scheme from 2019 to 2025 [49]) seeks to use digital health, particularly smart technology, in Vietnamese health care. The scheme highlighted the Industrial Revolution 4.0 concept and the technologies characterizing this era, for example, big data, AI, and the internet of things. Following the intention of the Vietnamese government to take advantage of this revolution, the scheme points out the readiness of Vietnam’s health system for implementing digital health technologies along with the impact that these technologies can have on the health system. Finally, the scheme presents an agenda to promote and implement digital and smart health IT in Vietnam. The information relevant to the scope of this study is summarized in the subsequent sections.

The scheme aims to *implement and develop digital health and smart health technologies for a modern, high-quality, equitable, efficient, and internationally integrated health system* and *to promote residents’ access to health information so that they can use a highly efficient health service and have their health continuously protected, taken care of, and promoted during their lifetime*. The following goals are specified:

1. Developing a smart health care and disease prevention system
2. Promoting IT implementation in health facilities for administrative process improvement and reducing hospital overload: adopting EMRs to replace paper records, using a cashless payment system for hospital billings, and establishing smart hospitals
3. Promoting IT implementation in health administration: installing the electronic office system, public portals, and single-window information system of administrative procedures, promoting level 3 and level 4 web-based public service, building a smart health administration

To achieve these goals, the scheme proposes 9 areas of action from 2019 to 2025, including the following:

1. Building the regulatory framework, guidance, standards, and economic–technical norms
2. Building the health IT infrastructure
3. Building a smart health care and disease prevention system
4. Building a smart health care system
5. Building a smart health administration system
6. Developing the workforce
7. Promoting smart health IT research, development, and implementation
8. International cooperation
9. Educating the public’s awareness of smart health care

In the scope of this study, we presented the detailed agenda of area 1—building the regulatory framework, guidance, standards, and economic–technical norms—and area 4, building a smart health care system (Textboxes 2 and 3).

**Area 1 of the Smart Health Information Technology Scheme: building the regulatory framework, guidance, standards, and economic–technical norms.**

**Area 1 of the Smart Health Information Technology Scheme**

- Building eHealth architecture framework as a prerequisite for information technology implementation in the health system
- Developing regulations for the resident health ID system
- Developing standards for interoperability between health information technology (HIT) systems, that is, health station management software, electronic medical records, and electronic health records (EHRs)
- Developing economic–technical norms for HIT, in which HIT costs are a part of the total health service cost
- Building policy for managing and using electronic nomenclature and coding systems in the health system
- Building policy for using EHRs
- Developing regulations for cybersecurity and privacy protection for health information in the web-based environment
- Building human resource policies for HIT specialists

**Area 4 of the Smart Health Information Technology Scheme: Building a Smart Health Care System.**

**Area 4 of the Smart Health Information Technology Scheme**

- Updating the management software and digital health systems in hospitals:
  Develop health information systems that adhere to the national and international standards, in which interoperability between the information systems and between the information system and the digital devices (lab devices, imaging diagnosis devices, interactive screens, personal mobile devices, etc) is ensured
  Standardizing the National Health ID system
  Building smart hospitals—health care organizations consult the Health Information Technology Maturity Model to develop their smart hospital roadmap
- Electronic medical records (EMRs) are implemented in all health care organizations according to the EMR rollout timeline in circular 46/2018/TT-BYT on the *Regulations for EMRs*, aiming for paperless medical records and cashless payments in hospitals
- Establishing and scaling up information kiosks in hospitals
- Promoting artificial intelligence (AI) application in health care with the following priorities:
  Building interoperability standards to implement the Internet of Medical Things as an infrastructure to operate clinical decision support systems (CDSSs)
  Developing real-time CDSSs closely integrated with EMRs
  Imaging diagnosis assistance
  Surgery assistance
  Encouraging businesses and health care organizations to build big data systems embedded with AI algorithm to support clinical decision-making
  Disease diagnosis, treatment, and prevention with traditional medicines
  Applying AI in specialties such as imaging diagnosis, cardiovascular diseases, respiratory diseases, orthopedics, cancer, obstetrics, and pediatrics

##### The Digital Transformation Scheme

The Digital Transformation Scheme (decision 5316/QD-BYT year 2020—The Digital Transformation in Health Care Scheme until 2025 and navigated toward 2030 [53]) seeks to implement IT in all aspects of health care as it defines digital transformation in health care as *the comprehensive application of information technology which prioritizes the cutting-edge digital technology that can make positive changes in all aspects of health care.*

The key 4 areas addressed in this scheme are state administration, cashless payment and telehealth, disease prevention and primary care, and health care. In the health care area, the scheme aims for 15% (210/1400) and 50% (700/1400) of hospitals in the country to successfully adopt paperless EMRs and cashless payment by 2025 and 2030, respectively.

Textbox 4 summarizes the areas and subareas of action set out in the scheme.

Area 5 (digital transformation in primary care and disease prevention) and area 6 (digital transformation in hospitals) are considered priorities over the others. We present details of area 1.2 (Textbox 5) and area 6 in subsequent sections.

Area 6, ie, Digital Transformation in Hospitals is largely similar to area 4 (building a smart health care system) of the Smart HIT Scheme. A comparison between these is shown in Table 5.

**Summary of areas of action in the Digital Transformation Scheme.**

**Areas of action**

- Area 1: infrastructure development
  Area 1.1: awareness education
  Area 1.2: building the regulatory framework, guidance, standards, and economic–technical norms
  Area 1.3: building and upgrading the information technology infrastructure
  Area 1.4: building health databases
  Area 1.5: building digital health platforms
  Area 1.6: ensuring cybersecurity
  Area 1.7: international cooperation, research, and innovations in digital health
  Area 1.8: workforce development
- Area 2: implementing information technology in administration and public services
- Area 3: promoting investments in digital health from businesses and hospitals
- Area 4: digitalization in societies
- Area 5: digital transformation in primary care and disease prevention
- Area 6: digital transformation in hospitals

**Area 1.2 of the Digital Transformation Scheme: building the regulatory framework, guidance, standards, and economic–technical norms.**

**Area 1.2 of the Digital Transformation Scheme**

- Publishing regulations and guidance for doing trials of novel digital health products; developing digital health platforms
- Developing regulations and standards for data exchange and interoperability between health information systems based on international standards
- Developing regulations for collecting and managing health data; building a decree for the national health database
- Providing guidance for digital health technologies; updating guidance for building smart hospitals and paperless hospitals
- Developing regulations for protecting security, safety, and confidentiality of health data in the web-based environment
- Developing regulations and guidance for electronic authentication in health care
- Implementing and updating the MoH’s e-government structure
- Developing financial mechanisms for health IT services as part of the overall health care service and mechanisms for hiring health IT services
- Developing policies and regulations for telemedicine and e-prescription so that patients can use remote health care services

**Table 5:** Comparing area 4 (decision 4888/QD-BYT year 2019) and area 6 (decision 5316/QD-BYT year 2020).

Category	Area 4 of the smart HIT^a^ scheme	Area 6 of the Digital Transformation Scheme
Updating management software and digital health systems in hospitals	Yes^b^	Yes
Standardizing the National Health ID system	Yes	Yes
Building smart hospitals based on circular 54/2017/TT-BYT	Yes	Yes
EMR^c^ implementation based on circular 46/2018/TT-BYT	Yes	Yes
Building information kiosks	Yes	No
Promoting AI^d^ application in health care	Yes	Yes
Conducting telehealth and web-based registration based on decision 2628/QD-BYT year 2020	No^e^	Yes
Implementing the national prescription management system in all health care organizations	No	Yes

^a^HIT: health information technology.

^b^The information was addressed in the document.

^c^EMR: electronic medical record.

^d^AI: artificial intelligence.

^e^The information was not addressed in the document.

#### EMRs: The Regulations for EMRs

EMR refers to the clinical record system used in hospitals’ inpatient departments. They are locally managed by health care institutions and different from the EHR system, which is centrally managed on the national database and intended for use by the commune health facilities and outpatient clinics (circular 46/2018/TT-BYT on *Regulations for EMRs* [50]).

This circular is the first official guidance for EMRs in public health care facilities. It ensures that the use of EMRs can uphold the equivalent law and can legitimately replace paper-based medical records. Health care organizations are eligible to discontinue paper-based medical record–keeping if they can satisfy all the criteria in this circular, which concern the following areas:

1. Content specifications
2. EMR creation and update
3. Storage and backup
4. Access right and secondary use
5. Patient identification
6. Digital or electronic signatures
7. Basic functionalities
8. Adherence to interoperability and IT standards
9. Data security and confidentiality
10. Nomenclature system

A total of 4 essential criteria that must be prioritized are as follows:

- Each patient’s EMR is provided with an ID number that is unique in the health organization.
- All information that a traditional medical record collects can also be recorded by EMRs.
- Each EMR must have a digital signature of the one who is responsible for the information entered in that record.
- Data stored in an EMR system are protected by Section 2, Article II of Cyber information Security Law [59].

In addition, the circular also provides the criteria for paperless lab test and imaging diagnosis management with LIS and picture archiving and communication system. Full details of this document can be found in Multimedia Appendix 2.

#### HIT Maturity: The HIT Maturity Model

##### Overview

The MoH put into effect the *HIT Maturity Model* (circular 54/2017/TT-BYT on Assessment Criteria for IT Implementation in Health Care Facilities [45]) as a roadmap for HIT implementation in health care facilities. Health care organizations are required to evaluate their current HIT maturity using this model and report the evaluation results to the MoH. The baseline results are a requisite for health care organizations to set goals for their next maturity level. An overall HIT maturity level depends on the maturity level of the individual domains, which are as follows:

1. IT infrastructure
2. Administration and operation software
3. HIS
4. RIS-PACS
5. LIS
6. Nonfunctionality standards
7. Security and information safety
8. EMR

In addition, there are specific capabilities that are required for each maturity level. An organization’s overall HIT maturity can range from *HIT level 1* to *HIT level* *7*.

There are 7 levels (1 to 7) applicable for the IT infrastructure domain and the HIS domain, and 2 levels (basic and advanced) applicable for the other domains (administration and operation software, LIS, nonfunctionality standards, security and information safety, RIS-PACS, and EMRs). The 7 levels of HIT maturity are summarized in subsequent sections. An overview of all the 7 levels can be found in Table 6.

**Table 6:** Levels of health information technology (HIT) maturity.

	Information technology infrastructure	HIS^a^	LIS^b^	RIS-PACS^c^	EMR^d^	Administration and operation software	Security and information safety	Nonfunctionality criteria	Extra capabilities
HIT level 7	Level 7	Level 7	Advanced	Advanced	Advanced	Advanced	Advanced	Advanced	“Paperless” hospital if all relevant criteria are met CDSS^e^ level 3 supporting doctors’ decisions related to treatment protocols and treatment results using suitably customized templates Data in CDR^f^ is analyzed to improve care quality, patient safety, and care efficiency Clinical data can be readily shared for stakeholders in care coordination based on HL7^g^ standards Continuous reports of hospital services using the data collected
HIT level 6 (smart hospital)	Level 6	Level 6	Advanced	Advanced	Basic	Advanced	Advanced	Advanced	CDSS level 2 providing: evidence-based warnings for treatment; that is, health and medicine advice, drug information and interaction check, and initial order and prescription violation identification rules All structured forms; that is, progress notes, consultation notes, problem lists, and discharge summaries are digitalized Closed-loop management of drugs, using identification technologies to assist drug administration
HIT level 5	Level 5	Level 5	Advanced	Advanced	N/A^h^	Basic	Basic	Basic	PACS^i^ can replace physical films
HIT level 4	Level 4	Level 4	Advanced	Basic	N/A	Basic	Basic	Basic	PACS allows doctors to access images outside the imaging department Electronic ordering Electronic management of inpatient orders
HIT level 3	Level 3	Level 3	Basic	N/A	N/A	Basic	Basic	Basic	Electronic records having digital vital sign records, nursing notes, medical procedures, and surgical procedures are stored in CDR CDSS level 1 assisting electronic prescription (new or historic prescription) Pharmacy information available in the hospital network and supported with CDSS
HIT level 2	Level 2	Level 2	N/A	N/A	N/A	N/A	N/A	N/A	A CDR consisting of normenclature and coding systems, pharmacy, orders, and test results (if available) Data in CDR can be shared between stakeholders for care coordination
HIT level 1	Level 1	Level 1	N/A	N/A	N/A	N/A	N/A	N/A	Patient’s information can be accessed electronically

^a^HIS: hospital information system.

^b^LIS: laboratory information system.

^c^RIS-PACS: radiology information system-picture archiving and communication system.

^d^EMR: electronic medical record.

^e^CDSS: clinical decision support system.

^f^CDR: clinical data repository.

^g^HL7: Health Level 7.

^h^N/A: not applicable.

^i^PACS: picture archiving and communication systems.

##### HIT Level 1

HIT level 1 includes level 1 of IT infrastructure and level 1 of HIS, which aims to provide electronic access to patient data. This IT infrastructure level essentially requires workstation computers, a local area network, and an internet connection while HIS manages outpatient and pharmacy data, and sends claim data to the health insurance claim system. Adoption of MoH’s terminology and service coding system is compulsory.

##### HIT Level 2

HIT level 2 is an upgrade of IT infrastructure and HIS from level 1 to level 2. This will add to the pre-existing system a dedicated server and the laboratory modules that manage laboratory orders and results. HIT level 2 particularly aims at building a clinical data repository (CDR) of pharmacy data, laboratory orders, test results, and the terminology and service coding system. Data in the CDR can be accessed by multiple stakeholders engaging in patient care.

##### HIT Level 3

HIT level 3 requires an upgrade of IT infrastructure and HIS from level 2 to level 3. Added components include LIS, administrative applications, and cybersecurity solutions at a *basic* level. At this level, more security and storage infrastructures are provided, including firewall devices, security solutions, and specialized storage. CDR capacity is increased to cover vital sign data, nurse notes, and medical procedures. Health care organizations would start implementing CDSS level 1 to assist doctors with drug information via the facility’s intranet.

##### HIT Level 4

HIT level 4 comprises level 4 IT infrastructure, level 4 HIS, advanced LIS, and a basic level of RIS-PACS, administration and operations software, cybersecurity, and nonfunctional standards. Organizations at this HIT level can manage their imaging diagnosis data electronically using RIS-PACS. Although a basic RIS-PACS has yet to discontinue physical film archiving [50], it offers essential functionalities such as integration with imaging diagnosis machines and HISs, digital image viewing, and measurement. An advanced LIS can manage test samples and supplies, integrate with the HIS, and send out alerts for abnormal test values. A storage network (a storage area network or a network-attached storage), a queue management system with display screens, will complement the IT infrastructure. Outpatient orders from doctors can be made electronically, whereas all inpatient orders will be managed in the digital system.

##### HIT Level 5

HIT level 5 differs from HIT level 4 in level 5 IT infrastructure, level 5 HIS, and *advanced RIS-PACS.* The advanced level of RIS-PACS is able to conduct multi-site consultation, allowing images to be viewed on multiple devices such as laptops and mobile phones. Furthermore, the *Regulations for EMRs* allow paperless image management if health care facilities have *advanced RIS-PACS* with an eligible storage capacity [50]. At level 5, HISs can manage emergency rooms, operating theaters, appointments, and follow-ups and can integrate electronic patient card.

##### HIT Level 6

Transitioning from HIT level 5 to HIT level 6 requires organizations to implement a *basic* EMR system in addition to upgrading their IT infrastructure, HIS, administration and operation software, security and data safety, CDSS, drug management, and nonfunctional standards to an *advanced* level. Hospitals qualified for HIT level 6 or higher are certified as a *smart hospital*. The basic EMR system provides inpatient medical records and can integrate with other clinical information systems. Mobile devices working on wireless local area network, hospital security cameras, and backup storage systems are needed to meet level 6 IT infrastructure.

Hospitals at HIT level 6 can receive and use data from multiple information systems such as drug information and interactions, treatment protocols, and nutrition. Clinical information can be accessed via mobile devices. In addition, HIT level 6 features several extra capabilities including a level 2 CDSS, a safe medicine management procedure, and increased digitalization of clinical documents. Level 2 CDSSs can warn doctors of drug interactions and potential treatment risks based on the most updated evidence. The medicine management system provides a closed and automatic medicine management procedure using automated dispensing systems and digital identification devices, for example, barcodes and radio frequency identification. All structured clinical forms such as progress reports, consultation notes, problem lists, and discharge summaries are digitized.

##### HIT Level 7

At HIT level 7, all 8 domains are at their highest level. Organizations are now equipped with information kiosks and network monitoring systems. A level 7 HIS provides functionalities to manage EMRs, professional procedures, control information kiosks, and support cashless payment. EMR systems at the *advanced* level have dedicated functionalities to manage medical records including increased storage duration, syncing records, and restoring records. Data stored in the EMR system are better protected and can be exchanged under international standards such as HL7. According to the *Regulations for EMRs* [50], an *advanced* EMR system with qualified storage capacity is eligible to replace the paper-based medical record system. Data from CDR are analyzed to improve quality of care, patient safety, and service efficiency. Insights for operational activities can be continuously generated to inform hospital departments such as inpatient, outpatient, and emergency departments. Organizations at this HIT level can exchange data using HL7 standards. Finally, level 3 CDSSs can support a wider range of diagnosis and treatment decisions (Table 6).

#### Conditions for Provision of Health IT: The Required Conditions for HIT

The Required Conditions for HIT (circular 53/2014/TT-BYT on Required conditions for provision of health IT activities [38]) can be seen as a summary of the criteria that health care facilities need to satisfy when implementing eHealth systems.

This circular announces the conditions and requirements that health care facilities operating in Vietnam must abide by when implementing and operating health IT systems. Health IT activities, as defined in the circular, involve *providing, transferring, collecting, analysing, storing and exchanging health care data through the IT infrastructure*. The conditions specify 4 areas related to health IT implementation: IT infrastructure, information security, human resource, and operational requirements (Multimedia Appendix 3).

#### General and Interoperability Standards

This group included 2 guidance documents, one from the MIC and one from the MoH. Although the former addressed the general technical standards that IT applications in state organizations need to adopt, the latter revolved around the national and international standards targeted at health information systems.

##### The Recommended Standards for HIT

In June 2013, the MoH published decision 2035/QD-BYT (year 2013—Terminology systems and data exchange standards recommended for health IT [36]) recognizing the terminology systems and technical standards applicable for health IT systems. Some terminologies and standards are required, whereas others are recommended for adoption in public health care organizations. It is strongly advised in the decision that all public facilities should adopt these systems, and information systems not matching them should plan for relevant transitions. Table 7 presents the standards and nomenclature systems recommended in the decision.

In addition to the standards, the MoH instructs IT systems in the health sector to follow other relevant IT standards from the MIC that were addressed in circular 01/2011/TT-BTTTT, which was then replaced by the new version, circular 22/2013/TT-BTTTT.

**Table 7:** Standards and nomenclature systems for health information technology systems.

Categories and standard names		Reference	Guidance
**Administrative nomenclature**			
	The list of official administrative units in Vietnam	Decision 124/2004/QD-TTg year 2004 and its amendments	Compulsory
	The list of ethnic groups in Vietnam	Decision121-TCTK/PPCD year 2004 and its amendments	Compulsory
	The list of occupations in Vietnam	Decision 114/1998/QD-TCTK year 1998 and its amendments	Compulsory
**International classification and coding for diseases and medical services**			
	ICD-10-CM^a^	WHO^b^	Compulsory
	ICD-O-3^c^	WHO	Recommended
	ICD-10-PCS^d^	WHO	Recommended
	ATC^e^	WHO	Recommended
	LOINC^f^	Regenstrief Institute	Recommended
**International interoperability standards**			
	Health Level 7 messaging version 2.x or 3.0	Health Level 7	Compulsory
	DICOM^g^ version 2.0	The National Electrical Manufacturers Association	Compulsory
	SDMX-HD^h^	WHO	Compulsory
	HL7 CDA^i^	Health Level 7	Recommended
	HL7 CCD^j^	Health Level 7	Recommended
	ELINCS^k^	The California HealthCare Foundation	Recommended

^a^ICD-10-CM: International Classification of Diseases, Tenth Revision, Clinical Modification.

^b^WHO: World Health Organization.

^c^ICD-O-3: International Classification of Diseases for Oncology, Third edition.

^d^ICD-10-PCS: International Classification of Diseases, Tenth Revision, Procedure Coding System.

^e^ATC: Anatomical Therapeutic Chemical.

^f^LOINC: Logical Observation Identifiers Names and Codes.

^g^DICOM: Digital Imaging and Communications in Medicine.

^h^SDMX-HD: Statistical Data and Metadata eXchange–Health Domain.

^i^HL7 CDA: Health Level 7 Clinical Document Architecture.

^j^HL7 CCD: Health Level 7 Continuity of Care Document.

^k^ELINCS: EHR-Lab Interoperability and Connectivity Specification.

##### The Recommended Standards for IT in State Organizations

The MIC released circular 39/2017/TT-BTTTT (Technical Standards for IT Implementation in State Organizations [46]) as guidance for the adoption of technical IT standards in state organizations. Its first version is circular 22/2013/TT-BTTTT, released in 2013. The circular advises on 5 categories of standards such as connection, data integration, information access, information security, and public key infrastructure. The MIC encourages systems currently not adopting the standards in this circular to carry out transitions by July 2018. A detailed list of the standards is shown in Multimedia Appendix 4.

#### Electronic Health Insurance Claim

##### The Insurance Portal V2 Guidance

**Figure 5:**
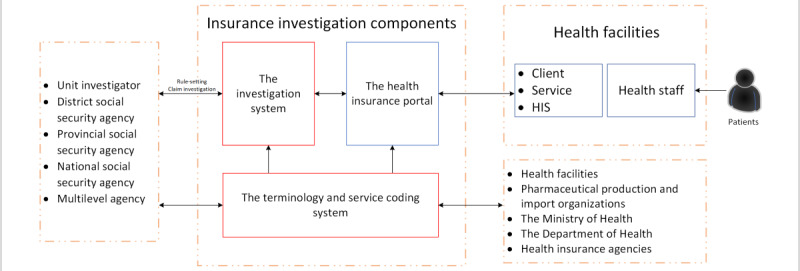
The Vietnam Social Security health insurance and investigation structure. HIS: health information system.

Decision 917/QD-BHXH year 2016 (Announcement of the health insurance portal version 2 [42]) from the VSS announces the health insurance portal version 2 and provides detailed guidelines on connection configuration and structuring data sets. The guideline is intended for use by health care facilities and provincial health insurance agencies who are responsible for regular data upload to the portal. The VSS health insurance claim and investigation structure are presented in Figure 5.

The guideline demonstrates 3 protocols that claim data can be submitted, each of which is accompanied by detailed instructions such as setting up connections, properly formatting data, and uploading data to the portal:

- Via web service connection—supports a variety of data submission including clinical data, service validation, monthly reports, health insurance ID checking, examination history, and referral receipt from other facilities.
- Data sync from client software—supports syncing clinical data to the system.
- Direct data entry on the portal—supports clinical data entry to the system.

The portal also offers functionalities for checking health insurance account information, viewing information of previous hospital visits, and checking referral documents between health care facilities.

##### The Electronic Claim Regulations

The MoH-mandated circular 48/2017/TT-BYT (Regulations on data exchange in management and reimbursement of health insurance claims [47]) as an official regulatory document for web-based health insurance claims and investigation for health care organizations. The circular essentially articulates the responsibilities of health care facilities and the health insurance agency as well as relevant rules in sending data and follow-ups. The fundamental technical requirements previously described in the VSS’s guidelines are also outlined in the document (Multimedia Appendix 5).

##### The Claim Standardization Guidance

The health insurance portal requires claim data to be formatted as XML, encoded with Unicode Transformation Format–8-bit (UTF-8), and relevant data sets to be standardized for efficient management and assessment. To support organizations in preparing claim data, the MoH has delivered data formatting guides and continuously updated them. The latest release is found in decision 4210/QD-BYT year 2017 (Requirements for standard and format of output data used in management, investigation and reimbursement of insurance-paid health care expenses [43]). Claims for groups of health services are recorded into separate data sets. Data sets from the same patient are linked through a unique ID number coded as *MA_LK*. The instructions include data item, data type, the allowed maximum length, and guidance for data collection.

The scope of the document is as follows:

- Guidance for claims of general health services
- Guidance for claims of medications
- Guidance for claims of medical procedures and supplies
- Guidance for claims of imaging diagnosis and laboratory test services
- Guidance for claims of follow-up interventions
- The official groups of services by cost
- The official hospital department codes
- The official accident and injury codes
- The official medicine bid packages and package codes

##### The Terminology and Service Coding System Version 6

The Service Coding System for Health Care Management and Health Insurance Reimbursement (decision 7603/QD-BYT year 2018 on the Service Coding System for Health Care Management and Health Insurance Reimbursement version 6 [48]), or the terminology and service coding system, was first piloted in 3 cities in Vietnam in 2015 [60]. Since then, it has been continuously updated, with version 6 [48] being the most up-to-date. The system features nomenclatures of health services, their standardized codes, regulated prices, and the ICD-10 codes with Vietnamese instruction. The terminology and service coding system has been made compulsory for use in health information systems of state health facilities and web-based health insurance claim activities. The 11 nomenclature groups provided in version 6 are as follows:

- Technical service codes
- Department codes
- Inpatient bed codes
- Half-day bed codes in chemotherapy and radiotherapy
- Medication codes
- Traditional medication codes
- Traditional diagnosis codes
- Medical supply codes
- Blood product codes
- ICD-10 codes
- Laboratory test codes

#### Cybersecurity in Health Organizations: The Cybersecurity Guidance

In October 2014, the MoH promulgated decision 4159/QD-BYT year 2014 (Guidance on ensuring the security of electronic health data in health organizations [37]) detailing the required measures to safeguard cybersecurity in health facilities. These regulations are applicable for organizations that conduct health IT activities in the web-based environment, including managing, using, storing, and exchanging health information. In general, the measures and recommendations addressed by this decision aim to maintain 3 characteristics (Textbox 6) in digital health implementation The 16 cybersecurity areas covered in the guidance can be found in Multimedia Appendix 6.

**Characteristics in digital health implementation safeguarded by The Cybersecurity Guidance.**

**Security**

- Only authorized users can access the health information.
- Passwords and access keys are encrypted during access and transmission and are saved at the health care facilities.

**Integrity**

- Information can only be deleted or edited by authorized users. Information is preserved during storing and transmission.
- Integrity is maintained in the management, use, storage, and transmission of information, in which changes are not allowed without authorization from the administration unit.
- Measures to ensure integrity must be applied in accessing, entering, storing, using, processing, transferring, extracting, and recovering data.

**Availability**

- Uninterrupted operation of the information technology (IT) system is ensured.
- Information can be accessed quickly in response to authorized requests.
- Human resource personnel operating the IT system is ensured.
- Policies for managing and implementing the IT system are developed, publicized, and adhered to (Multimedia Appendix 6).

#### LISs: The LIS Guidance

Decision 3725/QD-BYT year 2017 (guideline for implementation of LIS in health care facilities [44]) guides the technical features and functionalities of an LIS used in health care facilities.

The guideline first highlights the essential points that organizations need to consider when implementing or developing an LIS:

- LIS’s capability to communicate and integrate with other information systems in the hospital or health care system.
- Laboratory test results and related information can be exchanged and linked between different laboratories in a facility and with other facilities.
- LIS’s design and technical documents to allow for easy repair, maintenance, and upgrade.
- Technical standards recognized in circular 22/2013/BTTTT and decision 2035/QD-BYT year 2013, where applicable, must be adopted for LISs.
- Advanced technologies are encouraged for implementing LISs.
- Facilities must ensure the necessary conditions for managing and operating LISs such as safety, data security, IT infrastructure, and human resources (Multimedia Appendix 7).

#### HIMS: The HIMS Guidance

In 2006, the MoH released the decision 5573/QD-BYT year 2006 (Requirements and functional modules for hospital management software (HMS) [35]) to guide the adoption of HMS in Vietnam’s public and private health care facilities. The guideline addresses the key requirements that an HMS should meet in the regulatory and clinical context of Vietnam. An HMS’s functional modules and detailed instructions for each module are also presented in the document (Multimedia Appendix 8).

#### Electronic Health Records

##### Overview

The EHR system aimed to create and maintain a lifetime EHR for every Vietnamese resident. It differed from EMR systems that are managed by hospitals. EHR contents were developed based on the paper-based personal health record’s standardized template. Each EHR profile is identified by a unique health ID number.

##### The EHR Scheme

###### Overview

In decision 5349/QD-BYT year 2019 (The EHR Scheme [51]), the MoH presents the scheme for setting up and implementing the EHR system on a national scale. Accordingly, an EHR is the digital version of health records that are created, displayed, updated, stored, and exchanged using electronic devices. Health records are the medical documents that keep health care information of a person for their lifetime and are regulated by the MoH. The ultimate goal of this scheme is to provide every Vietnamese citizen with an EHR while step-by-step establishing a population health database in the National Health Data Center.

The scheme requires the manufacturers to apply specific standards for the EHR software. Textbox 7 lists the requirements for EHR systems according to the EHR Scheme.

Every EHR profile will have a national health ID number that is unique for every citizen. The National Health ID system is guided by decision 2153/QD-BYT year 2020 [54].

For data ownership, the government’s administrative bodies (the MoH and the relevant Provincial Department of Health) own and manage the data generated during EHR use. EHR providers or EHR developers have responsibilities to hand over data, software source codes, and other tools that allow the MoH or the Department of Health to continue the EHR service with another provider.

**Summary of requirements for electronic health record systems.**

**Category and requirements**

- The electronic health record (EHR) software’s design must meet the required standards:
  The EHR software can collect all the data in a standard paper health record guided in decision 831/QD-BYT year 2017 (the standard template of personal health record for primary health care)
  The EHR software can export data to XML files that follow the guidance set out in decision 4210/QD-BYT year 2017 (Standards for claim data submitted to the web-based health insurance system)
  The EHR software can satisfy the regulations in circular 48/2017/TT-BYT year 2017 (regulations for data sharing in health insurance claim)
  Compatible with Health Level 7 standards
  The EHR software can use the National Health ID system and is interoperable with related health information systems
- EHR functional modules
  Health service provision functionality
  Administrative management functionality
  Information infrastructure management functionality
- Personal information protection
  Protection of personal data in EHRs abides by Section 2, Chapter II of the Electronic Data Safety Law

Per the timeline announced in the scheme, the MoH aims to reach an EHR coverage of 80% in the population of central government provinces by the end of 2020 and a coverage of 95% in Vietnam’s population by the end of 2025. Following are the steps to install and implement EHRs:

- Developing EHR software
- Installing EHR software in health facilities
- Providing EHR training for health workers
- Creating EHRs and collecting data for EHRs using the pre-existing data at health facilities or via interviews
- Continuously updating data to EHRs at health facilities
- Making use of data collected in EHRs
- Maintaining the EHR system

Technical documents and guidance to operate and manage EHRs will be built to guide EHR use at health facilities, including the following:

- Interoperability specifications with the National Health ID system and related health information systems.
- Policies for using, managing, operating, and ensuring data safety for the EHR system.
- Policies for creating, updating, and making use of EHRs.
- Financial mechanisms for maintaining and operating the EHR system.

##### The Health ID Regulation

Decision 2153/QD-BYT year 2020 (Regulations on creation, use, and management of health identification [54]), released in May 2020, announced that the social insurance ID will be the national health ID. These ID numbers are nationally unique for each patient and represent a set of identification data for that patient. Each identification data set includes the patient’s full name, date of birth, gender, place of birth ID, and health insurance ID. Health ID is intended to be used with EHR, EMR, and other health information systems as a common patient identifier system.

## Discussion

### Principal Findings

This review explored the current state of digital health research and policies in Vietnam to inform the implementation of digital systems used in hospital care. Nearly half of the hospital-based studies were case reports of engineering solutions; 1 study assessed physicians’ performance with assistance from a CDS software; 2 explored readiness to adopt EHRs; and 2 provided a high-level review of Vietnamese eHealth. The data analyzed in these studies were mostly collected in 2016 or earlier. These findings suggest a lack of research studies that investigate and inform the implementation of digital health systems in Vietnamese hospital settings.

Government policies in Vietnam have paid significant attention to digital health over the past 5 years. The MoH has set out specific regulations for EMR use and a maturity framework that shapes the development of digital health systems in hospitals. Other national projects have occurred, such as the electronic insurance claim system, the nationwide rollout of an EHR, and a national health ID system. International standards such as HL7v2 messaging, ICD-10, and recently HL7’s Fast Healthcare Interoperability Resources have been consistently recommended. Guidance documents have also addressed measures to ensure cybersecurity in health organizations. These policies are guided by an architectural framework and digital health strategies that ultimately aim to leverage IT, especially smart technology, in all aspects of the sector.

Of all the policies reviewed, the *Regulations for EMRs* and the *HIT Maturity Model* can be seen as the key policies for HIT application in Vietnam’s hospitals. Although the previous national efforts focused on administrative systems such as the HIMS [35], these policies aimed to promote the adoption of clinical systems such as EMR, laboratory information management system, and RIS-PACS. At this nascent stage of HIT adoption, Vietnam has been focusing on building the necessary infrastructure and emphasizing the role of HIT in achieving a more efficient paperless environment. This is reflected in the recent digital health agenda, which highlighted the crucial role of EMRs in replacing paper medical records [49,53]. A major theme that the Regulations for EMRs addressed is the eligibility of transitioning to a completely paperless environment for EMR systems, as well as LIS and RIS-PACS [50]. Although this is a practical and well-defined motivation for EMR adoption, the World Health Organization recommended the true benefits of EMRs, such as improved data quality, timely access to information, and increased care quality, should be proven to encourage buy-in from health care workers [61]. Thus, future policies may seek to link EMR and HIT adoption with such benefits through clearly defined quality indicators to ensure meaningful HIT use. Lessons learned from some countries also recommend that financial incentives, when possible, should be made to increase EMR adoption [62-64].

The *HIT Maturity Model* defined a wide spectrum of HIT implementation levels that are applicable for hospitals of various digitization stages. Although the *Smart HIT Scheme* and the *Digital Transformation Scheme* aimed to establish smart hospitals (HIT level 6) in the next few years, there may be a need for further policy development that takes into account the Vietnamese context as an LMIC. The *Assessment of HIT Maturity* conducted in 2019 showed a large dispersion of HIT maturity across the top-level hospitals directly governed by the MoH, among which 19% (8/42) were only at HIT level 1, most hospitals (20/42, 48%) were at HIT level 3, and only 2% (1/42) of hospitals was at HIT level 5 [65]. A readiness assessment conducted by the MoH in 2017 revealed that among 36 MoH-governed hospitals, only 11 (31%) had EMR systems, 14 (39%) had some forms of CDS software, and over half of these hospitals lacked specialized IT workforce and information security capacity [66]. Given the low HIT maturity levels that these high-rank hospitals held, HIT implementation and readiness in smaller hospitals can be even poorer. It is likely that rural hospitals will struggle to reach even the most basic levels of digital maturity owing to a lack of funding, IT workforce, and infrastructure. Therefore, future policies may need to recommend further support for small and rural hospitals to reduce these gaps in digital maturity.

The 2 digital health strategies aim to promote AI in health care through applications such as CDSSs integrated with EHR systems, imaging diagnosis, and surgical support. New regulations, guidance, and standards are to be released to inform these adoptions [53]. Some of these intended policies are regulations for trials of novel digital health products, guidance for digital health technologies, and guidance for protecting web-based health data, to name a few. New regulation frameworks, along with an increased use of EMRs and EHRs in Vietnam’s hospitals, will be important facilitators for the development and implementation of AI for hospital care in Vietnam. However, there is currently a lack of research to inform appropriate and sustainable use of these technologies in Vietnam. Local context factors such as disease burden, health care practices, and infrastructure constraints should be considered for future AI research and implementation, as highlighted by Schwalbe and Wahl [5], Wahl et al [6], and Alami et al [67]. Future digital health research should seek to investigate these factors to best guide HIT and AI development and application in Vietnam’s hospitals.

### Limitations

This review was limited to the context of hospital-based digital health systems and does not cover the more extensive academic literature on community-based systems and the use of national systems for monitoring infectious disease outbreaks and other public health interventions. Academic studies were only searched on international databases, hence this study did not cover publications in Vietnamese journals that are not registered in these databases.

At the time this study was conducted, the digital health policy landscape in Vietnam was experiencing significant changes with new policies likely to be released and the existing policies being subject to modifications over the next year. Therefore, this scoping review should be viewed as a snapshot of this area at the end of 2020.

### Conclusions

Quantity and areas of research studies about digital health systems in hospitals in Vietnam are limited with little evidence to inform the implementation of new technologies in hospital care. The policies developed over the past 5 years to inform the adoption of digital health systems in Vietnam are comprehensive and will be useful for hospitals. They focus on guiding the basic functionalities and largely follow international standards and guidelines. Further research is needed to ensure that policies and guidelines can deal in detail with issues not encountered in HIC settings or that may be specific to the Vietnamese context.

## Appendix

Multimedia Appendix 1 Literature search strategies.

Multimedia Appendix 2 Circular 46/2018/TT-BYT on regulations for electronic medical records.

Multimedia Appendix 3 Circular 53/2014/TT-BYT on required conditions for provision of health information technology activities.

Multimedia Appendix 4 Circular 39/2017/TT-BTTTT on technical standards for information technology implementation in state organizations.

Multimedia Appendix 5 Circular 48/2017/TT-BYT on regulations on data exchange in management and reimbursement of health insurance claims.

Multimedia Appendix 6 Decision 4159/QD-BYT year 2014 on guidance on ensuring security of electronic health data in health organizations.

Multimedia Appendix 7 Decision 3725/QD-BYT year 2017 on guideline for implementation of laboratory information system in health care facilities.

Multimedia Appendix 8 Decision 5573/QD-BYT year 2006 on requirements and functional modules for hospital management software.

## Abbreviations

AI: artificial intelligence
CDR: clinical data repository
CDS: clinical decision support
CDSS: clinical decision support system
EHAF: eHealth architectural framework
EHR: electronic health record
EMR: electronic medical record
HIC: high-income country
HIMS: Hospital Information Management System
HIS: hospital information system
HIT: health information technology
HL7: Health Level 7
HMS: hospital management software
ICD-10: International Classification of Diseases, Tenth Revision
IT: information technology
LIS: laboratory information system
LMIC: low- and middle-income country
MIC: Ministry of Information and Communications
MoH: Ministry of Health
PRISMA-ScR: Preferred Reporting Items for Systematic Reviews and Meta-Analyses Extension for Scoping Reviews
RIS-PACS: radiology information system and picture archiving and communication systems
SDMX-HD: Statistical Data and Metadata Exchange–Health Domain
UTF-8: Unicode Transformation Format–8-bit
VSS: Vietnam Social Security
