# Supplementary material for: Digital Health Policy and Programs for Hospital Care in Vietnam: Scoping Review
Source: J Med Internet Res. 2022 Feb 9;24(2):e32392. doi: 10.2196/32392 (PMC8867296; doi:10.2196/32392)
Supplement: Multimedia Appendix 1 [file jmir_v24i2e32392_app1.doc]

## Multimedia Appendix 1. Literature search strategies.

**Search Strategy**

Governments’ policies and programmes were searched on thuvienphapluat.vn database using the following search terms:

1. "-BYT" "điện tử" (search date: March 5^th^ 2020; search in title)

2. "-BYT" "bệnh án" (search date: March 5^th^ 2020; search in title)

3. "-BYT" "công nghệ" (search date: March 5^th^ 2020; search in title)

4. "-BYT" "thông tin" (search date: March 5^th^ 2020; search in title)

5. "-BYT" "thông minh" (search date: March 5^th^ 2020; search in title)

6. "-BYT" "dữ liệu" (search date: March 5^th^ 2020; search in title)

7. "-BYT" "trực tuyến" (search date: March 5^th^ 2020; search in title)

8. "-BYT" "mạng" (search date: March 5^th^ 2020; search in title)

9. "-BYT" "phần mềm" (search date: March 5^th^ 2020; search in title)

10. "-BYT" "thiết bị" (search date: March 11^th^ 2020; search in title)

*English explanation for search terms:

**BYT**: code for offical documents from the MoH (BYT is Vietnamese abbreviation of Ministry of Health); **điện tử** = electronic; **bệnh án** = health records; **công nghệ** = technology; **thông tin** = information; **thông minh** = smart; **dữ liệu** = data; **trực tuyến** = online; **mạng** = internet; **phần mềm** = software; **thiết bị** = devices.

**Final search strategy for Pubmed search (performed on March 23^rd^ 2020):**

("Medical Informatics"[58] OR (health* AND informatic*) OR ((computerised OR digital OR electronic) AND health*) OR ehealth OR “e-health” OR (health* AND (“information system” OR information technolog* OR "information management system")) OR EHR OR EHRs OR EMR OR EMRs OR ((computerised OR electronic OR digital) AND (health record* OR patient record* OR medical record*))) AND (Vietnam OR “Viet Nam”)

**Web of Science search was conducted by a librarian at the Bodleian Health Care Libraries based on the final Pubmed search queries.**
