# Supplementary material for: Digital Health Policy and Programs for Hospital Care in Vietnam: Scoping Review
Source: J Med Internet Res. 2022 Feb 9;24(2):e32392. doi: 10.2196/32392 (PMC8867296; doi:10.2196/32392)
Supplement: Multimedia Appendix 2 [file jmir_v24i2e32392_app2.doc]

## Multimedia Appendix 2

**Circular 46/2018/TT-BYT on Regulations for electronic medical records**

**Content specifications of EMR**

The content and classification specifications for records entered into EMR systems are based on the current regulations and MoH guidelines for medical records in Vietnam. Accordingly, EMRs are classified into inpatient record, outpatient record, and other specialized records. EMRs must be able to capture all the data that are normally collected in the MoH standard medical record templates. The relevant guidelines for EMR formats are shown below.

| Decision 4069/2001/QD-BYT | Standard formats for medical records and documents. |
| --- | --- |
| Circular 50/2017/TT-BYT | Amendments for some regulations on healthcare cost payment. |
| Decision 4069/QD-BYT year 2010 | Standard formats for traditional medicine records. |
| Decision 999/QD-BYT year 2011 | Standard formats for abortion records. |
| Decision 3443/QD-BYT year 2011 | Standard formats for ophthalmology records. |
| Decision 1456/QD-BYT year 2012 | Standard formats for hand-foot-and-mouth disease records and some related regulations. |

**EMR creation and update**

The maximum duration for updating information in an EMR system should be no longer than 12 hours from when the doctor gave their clinical decisions, or 24 hours if the clinical examination lasts longer than 12 hours or an IT issue occurs.

**EMR storage and backup**

The circular states that archiving paper medical records can be discontinued following EMR implementation if the following four criteria are met:

1. The EMR system has reached the “Advanced” level as defined in Circular 54/2017/TT-BYT (Assessment Criteria for Information Technology Implementation in Healthcare Facilities).
2. The system’s storage is capable of storing EMR data for the time span regulated in the healthcare law (10 years for outpatient/inpatient records; 15 years for accident records; and 20 years for mental disorders and mortality records).
3. Backup of EMR data is completed weekly and the backup data are saved at a data centre that satisfies the requirements from the Ministry of Information and Communication.
4. Organizations will transfer their EMR data to the new owner organization in case of mergers or dissolution.

**Access right and secondary use**

These regulations define the different groups that can request for access to information in EMRs, how the information can be accessed, and the restrictions for each group. These authorized groups were originally defined in the Healthcare Law, which regulates access to paper medical records. There are three groups with different levels of EMR access as follows:

- Staff of the healthcare facility, healthcare students, and researchers are able to view EMR data on-site or make an electronic copy of EMR contents for research or professional purposes.
- Representatives from the supervising agency of the healthcare facility, inspectors, procuracy, court officials, health inspectors, health insurance agencies, forensic agencies and lawyers can view EMR data on-site or make an electronic/printed copy of the EMR contents after being authorized by the head of the healthcare facility.
- The patient or their representatives can send a written request for their electronic or paper medical record summaries. EMR summaries must cover all the information listed in the standard medical record summary template (Appendix 4 of Circular 56/2017/TT-BYT on Detailed Guidance for Health-related Sections in Social Security Law and Hygiene and Labour Safety Law). Accordingly, information required in the medical record summary includes patient’s name, demographics information, social insurance/health insurance ID, hospital admission/discharge information, and summaries of clinical progress, important test results, treatment, and discharge status.

All requests for access must be approved by the organization’s authorities in charge. People who have authorization to access and use EMRs must ensure confidentiality and comply with the permitted rights.

**Patient identification**

Patient IDs follow the common national health ID system announced by the MoH.

**Digital/electronic signatures**

- Digital or electronic signatures are used to authenticate the health professional, the patient or their representatives who are responsible for the information on the EMR.
- When records are originally signed with electronic signatures, the healthcare facility must certify these signatures by an authorized digital signature.
- Organizations is required to develop and implement local policies on digital and electronic signatures in prior to their implementation.
- During the transition from paper-based medical records to EMRs, there are some paper documents that do not have a standard digital template and are unable to be signed with digital signatures. A workaround is allowed in which the document will be manually signed, scanned, and digitalized to be attached to the EMR profile, while the original document must be archived.

**EMR functionalities**

The EMR policy states that EMRs must comply with the following basic requirements:

- Use the MoH’s terminology and service coding system and apply the health IT standards recommended in this circular.
- The EMR system must have user control capabilities including user authentication and authorization, an audit trail, and capabilities to protect confidentiality and security.
- The EMR system must be able export data to XML files customized to the following purposes:
  - Medical record summaries (as defined in Circular 56/2017/TT-BYT).
  - Primary health records (as defined in Decision 831/QD-BYT year 2017).
  - Sharing claim data to the health insurance portal (as defined in Decision 4210/QD-BYT year 2018).
- Data in EMRs can be displayed on computer screens or other devices
- Data in EMRs can be exported to the standard layouts of paper medical records and can be printed.
- Adhere to the functional modules defined in Circular 54/2017/TT-BYT (e.g., healthcare service management, administrative management, and medical record management).

**Adherence to health IT standards**

The circular recommends the EMR systems adopt specific health IT standards, including HL7 CDA, HL7 FHIR, DICOM version 2.0 or later, and relevant IT standards recommended by the Ministry of Health and the Ministry of Information and Communication.

**Data security and confidentiality of EMRs**

The circular requires that healthcare facilities have measures in place to safeguard the security and confidentiality of data stored in EMRs that suit the EMR system’s specifications and organization’s policies:

- The regulations on access rights and secondary use of EMR data described in this circular, and other relevant regulations from the MoH must be imposed in the facility.
- The facility must have solutions and protocols in place to protect cybersecurity during EMR implementation, including:
  - Access control solutions, including user authentication and authorization, and setting access time.
  - Preventing unauthorized access.
  - Restoring data lost in accidents.
  - Preventing, detecting and stopping malware.
- Data must be encrypted when being shared between facilities.
- Data must be encrypted based on relevant MoH guidelines.
- The EMR system must have audit trail functionality that can track data and time of activities such as viewing, submitting new data, editing, removing, and restoring data.
- Each facility must have their local policies and regulations on data security and patient privacy based on the current laws and guidelines.

**Nomenclature system**

The MoH’s terminology and service coding system must be used to standardize data in the EMR system.

**Criteria for LIS and PACS to discontinue physical film and paper-based archiving**

The circular also sets out the criteria for LIS and PACS to discontinue keeping physical films and paper-based lab result registries. Accordingly, LIS and PACS implementation must reach Advanced level in Circular 54/2017/TT-BYT while their storage capacity can satisfy the archiving time similar to that of EMR systems.
