# Supplementary material for: Digital Health Policy and Programs for Hospital Care in Vietnam: Scoping Review
Source: J Med Internet Res. 2022 Feb 9;24(2):e32392. doi: 10.2196/32392 (PMC8867296; doi:10.2196/32392)
Supplement: Multimedia Appendix 3 [file jmir_v24i2e32392_app3.doc]

Multimedia Appendix 3. Circular 53/2014/TT-BYT on required conditions for provision of health information technology activities.

**Operational Requirements for Health IT Systems**

**Circular 53/2014/TT-BYT – Required conditions for provision of health IT activities**

IT infrastructure

These criteria make sure healthcare facilities have appropriate infrastructure for efficient and secured service delivery. There are four domains covered: server and system software, network system, database and workstations. Specific requirements are presented in the table below.

| Server and system software | Server infrastructure and associated equipment has sufficient performance, efficiency, data processing speed and data retrieval speed for the provision of online healthcare service |
| --- | --- |
|  | The server has high availability and flexible backup mechanism for interrupted operation. |
|  | The system software and operation system have legal license and transparent origin. |
| Network system | Network systems such as telecommunication network, Internet, wide area network, local area network and other connections are appropriately designed and implemented, with capable bandwidth. The telecommunication network must meet the requirements in Article 16 of Telecommunication Law. |
|  | Network devices and network management software have legal license and transparent origin. |
|  | Backup plan is pre-built to ensure network operation. |
| Database | Database for the online healthcare services must be stable and able to effectively process and store the data created during operation. |
|  | Use a proprietary management system with a legal license and transparent origin, or open-source management system widely used nationally or worldwide. |
| Workstation | Decent number of workstations with appropriate configuration to operate the health IT services. |

Information security

These are the requirements that help organizations to maintain system’s security and protect their data from unauthorized access or accidents. Key solutions include cybersecurity policy, network security, application software security, data safety, and issue management protocols. Details for each solution are shown below.

| Have SOPs for issue management | Have issue management SOPs that address personnel's responsibilities and detail solving steps including notifying the users and administrators. If the IT service is purchased externally, the provider must include these SOPs in the service package. |
| --- | --- |
| Issue check and management | Conduct frequent issue checks, update information about new issues and their management procedures |
| Cyberattack prevention | Employ techniques to timely detect and prevent cyber attacks |
| Proactive IT disaster prevention | Apply solutions to prevent IT risks and disasters to maximally limit cyber risks related to health IT activities |

Human resource

Requirements for specialized IT staff in healthcare facilities are also addressed in this circular. These criteria examine professional competency, number of staff and routine trainings. In general, facilities at higher administrative levels need to meet higher standards.

| Staff competency and quantity | Ensure there are enough IT specialized staff with competency. |
| --- | --- |
| Requirement for facilities at special level or level 1 | These facilities must have IT department with at least 5 staff, of which at least 60% have Intermediate Degree or higher. |
| Requirement for facilities at level 2 or level 3 | These facilities must at least have an IT group with at least 3 staff with Associate Degree or higher. |
| Training for health IT staff | Have training plans and organize trainings for health IT staff. |
| Hiring of external staff | Make sure the hired external staff have competency for the work. The contract must require adherence to healthcare law in protecting patients’ privacy and confidentiality. |

Requirements for specific heath IT services

In addition to the aforementioned IT conditions, health facilities need to follow particular standards and requirements during delivering their health IT services.

- Relevant procedures should be standardized so health IT applications can be implemented effectively.
- Health IT systems and applications should apply the following national and international standards:

| HL7 standards (HL7 messaging 2.x, HL7 messaging 3.0, HL7 CDA) |
| --- |
|
| DICOM (Digital Imaging and Communications in Medicine) |
| ISO/IEEE 11073 (a standard for medical device communication) |
| SDMX-HD (Indicators and metadata exchange standard) |
| IT standards recognized in Circular 22/2013/TT-BTTTT by the Ministry of Information and Communication |

- Each healthcare organization must develop and enact their IT management and implementation policy.
- Secondary use of patient data must ensure patient’s privacy and confidentiality regulated by healthcare law.
- Using digital signatures and digital identifications are allowed and regulated by Decree 26/2007/ND-CP, Decree 106/2011/ND-CP, Decree 170/2013/ND-CP, and Decree 106/2011/ND-CP.
- EMR creation, storage, and implementation must adhere to article 59 of healthcare law.

When a healthcare facility employs an IT service from an external provider, the contracts and terms must address data ownership and relevant responsibilities when issues happen.
