# Supplementary material for: Digital Health Policy and Programs for Hospital Care in Vietnam: Scoping Review
Source: J Med Internet Res. 2022 Feb 9;24(2):e32392. doi: 10.2196/32392 (PMC8867296; doi:10.2196/32392)
Supplement: Multimedia Appendix 4 [file jmir_v24i2e32392_app4.doc]

## Multimedia Appendix 4

**Circular 39/2017/TT-BTTTT on Technical standards for IT implementation in state organizations**

| **Type of standard** | **Standard code** | **Full name of standard** | **Application** |
| --- | --- | --- | --- |
| **Connection standards** | | | |
| Hypertext transfer | HTTP v1.1 | Hypertext Transfer Protocol version 1.1 | Compulsory |
|  | HTTP v2.0 | Hypertext Transfer Protocol version 2.0 | Recommended |
| File transfer | FTP | File Transfer Protocol | Compulsory application of one or both standards |
|  | HTTP v1.1 | Hypertext Transfer Protocol version 1.1 |  |
|  | HTTP v2.0 | Hypertext Transfer Protocol version 2.0 | Recommended |
|  | WebDAV | Web-based Distributed Authoring and Versioning | Recommended |
| Audio/ image streaming and transport | RTSP | Real-time Streaming Protocol | Recommended |
|  | RTP | Real-time Transport Protocol | Recommended |
|  | RTCP | Real-time Control Protocol | Recommended |
| Data access and sharing | OData v4 | Open Data Protocol version 4.0 | Recommended |
| Mail transfer | SMTP/ MIME | Simple Mail Transfer | Compulsory |
|  |  | Protocol/Multipurpose |  |
|  |  | Internet Mail Extensions |  |
| Provision of internet message access service | POP3 | Post Office Protocol version 3 | Compulsory application of both standards to the server |
|  | IMAP 4rev1 | Internet Message Access Protocol version 4 revision 1 |  |
| Directory access | LDAP v3 | Lightweight Directory Access Protocol version 3 | Compulsory |
| Domain name service | DNS | Domain Name System | Compulsory |
| Connection-oriented transport | TCP | Transmission Control Protocol | Compulsory |
| Connectionless transport | UDP | User Datagram Protocol | Compulsory |
| LAN/WAN internetwork | IPv4 | Internet Protocol version 4 | Compulsory |
|  | IPv6 | Internet Protocol version 6 | Compulsory application of this standard to Internet-connected equipment |
| Wireless Local Area Network | IEEE 802.11g | Institute of Electrical and Electronics Engineers Standard (IEEE) 802.11g | Compulsory |
|  | IEEE 802.11n | Institute of Electrical and Electronics Engineers Standard (IEEE) 802.11n | Recommended |
| Wireless Internet access | WAP v2.0 | Wireless Application Protocol version 2.0 | Compulsory |
| SOAP web service | SOAP v1.2 | Simple Object Access Protocol version 1.2 | Compulsory application of one, two or three standards |
|  | WSDL V2.0 | Web Services Description Language version 2.0 |  |
|  | UDDI v3 | Universal Description, Discovery and Integration version 3 |  |
| RESTful web service | RESTful web service | Representational state transfer | Recommended |
| Web services specifications | WS BPEL v2.0 | Web Services Business Process Execution Language Version 2.0 | Recommended |
|  | WS-I Simple SOAP Binding Profile Version 1.0 | Simple SOAP Binding Profile Version 1.0 | Recommended |
|  | WS- Federation v1.2 | Web Services Federation Language Version 1.2 | Recommended |
|  | WS- Addressing v1.0 | Web Services Addressing 1.0 | Recommended |
|  | WS-Coordination Version 1.2 | Web Services Coordination Version 1.2 | Recommended |
|  | WS-Policy v1.2 | Web Services Coordination Version 1.2 | Recommended |
|  | OASIS Web Services Business Activity Version 1.2 | Web Services Business Activity Version 1.2 | Recommended |
|  | WS- Discovery Version 1.1 | Web Services Dynamic Discovery Version 1.1 | Recommended |
|  | WS- MetadataExchange | Web Services Metadata Exchange | Recommended |
| Network time service | NTPv3 | Network Time Protocol version 3 | Compulsory application of one of the two standards |
|  | NTPv4 | Network Time Protocol version 4 |  |
| **Data integration standards** | | | |
| Extensible Markup Language | XML v1.0 (5^th^ Edition) | Extensible Markup Language version 1.0 (5^th^ Edition) | Compulsory application of one of the two standards |
|  | XML v1.1 (2^nd^ Edition) | Extensible Markup Language version 1.1 |  |
| Electronic Business Extensible Markup Language | ISO/TS 15000:2014 | Electronic Business | Compulsory |
|  |  | Extensible Markup |  |
|  |  | Language (ebXML) |  |
| XML Schema Definition | XML Schema V1.1 | XML Schema version 1.1 | Compulsory |
| Data transformation | XSL | Extensible Stylesheet Language | Compulsory application of the latest version |
| Object modelling | UML v2.5 | Unified Modelling Language version 2.5 | Recommended |
| Resource description | RDF | Resource Description Framework | Recommended |
|  | OWL | Web Ontology Language | Recommended |
| Character set demonstration | UTF-8 | 8-bit Universal Character Set (UES)/Unicode Transformation Format | Compulsory |
| Geographic information exchange format | GML v3.3 | Geography Markup Language version 3.3 | Compulsory |
| Geographic information access and update | WMS v1.3.0 | OpenGIS Web Map Service version 1.3.0 | Compulsory |
|  | WFS v1.1.0 | Web Feature Service version 1.1.0 | Compulsory |
| XML metadata interchange specification | XMI v2.4.2 | XML Metadata Interchange version 2.4.2 | Recommended |
| Metadata registries (MDR) | ISO/IEC 11179:2015 | Metadata registries - MDR | Recommended |
| Dublin Core metadata element set | ISO 15836- 1:2017 | Dublin Core metadata element set | Recommended |
| JavaScript Object Notation (JSON) Data Interchange Format | JSON RFC 7159 | JavaScript Object Notation | Recommended |
| Business Process Modelling Language | BPMN 2.0 | Business Process Model and Notation version 2.0 | Recommended |
| **Information access standards** | | | |
| Web content | HTML v4.01 | Hypertext Markup Language version 4.01 | Compulsory |
|  | WCAG 2.0 | W3C Web Content Accessibility Guidelines (WCAG) 2.0 | Recommended |
|  | HTML 5 | Hypertext Markup Language version 5 | Recommended |
| Extensible Web content | XHTML v1.1 | Extensible Hypertext Markup Language version 1.1 | Compulsory |
| User interface | CSS2 | Cascading Style Sheets Language Level 2 | Compulsory application of one of the three standards |
|  | CSS3 | Cascading Style Sheets Language Level 3 |  |
|  | XSL | Extensible Stylesheet Language version |  |
| Document | (.txt) | Plain Text (.txt) format: for unstructured documents | Compulsory |
|  | (.rtf) v1.8, v1.9.1 | Rich Text (.rtf) format v1.8, v1.9.1: for cross-platform document interchange | Compulsory |
|  | (.docx) | Microsoft Word open document format (.docx) | Recommended |
|  | (.pdf) v1.4, v1.5, v1.6, v1.7 | Portable Document (.pdf) format v1.4, v1.5, v1.6, v1.7: for read-only documents | Compulsory application of one, two or three standards |
|  | (.doc) | Microsoft Word document format (.doc) |  |
|  | (.odt) v1.2 | Open Document Text (.odt) v1.2 |  |
| Spreadsheets | (.csv) | Comma separated Variable/Delimited (.csv) format: for  exchanging data between different applications | Compulsory |
|  | (.xlsx) | Microsoft Excel open XML spreadsheet file format (.docx) | Recommended |
|  | (.xls) | Microsoft Excel spreadsheet file format (.xls) | Compulsory application of one or both standards |
|  | (.ods) v1.2 | Open Document Spreadsheets file format (.ods) v1.2 |  |
| Demonstration | (.htm) | Hypertext Document (.htm) format: for presentations exchanged through different types of browser | Compulsory |
|  | (.pptx) | Microsoft Open PowerPoint file format (.pptx) | Recommended |
|  | (.pdf) | Portable Document (.pdf) format: for read-only presentations | Compulsory application of one, two or three standards |
|  | (.ppt) | Microsoft PowerPoint (.ppt) file format |  |
|  | (.odp) v1.2 | Open Document Presentation (.odp) file format v1.2 |  |
| Graphic image | JPEG | Joint Photographic Expert Group (.jpg) | Compulsory application of one, two, three or four standards |
|  | GIF v89a | Graphic Interchange (.gif) version 89a |  |
|  | TIFF | Tag Image File (.tif) |  |
|  | PNG | Portable Network Graphics (.png) |  |
| Georeferenced image | GEO TIFF | Tagged Image File Format for GIS applications | Compulsory |
| Moving picture, audio | MPEG-1 | Moving Picture Experts Group-1 | Recommended |
|  | MPEG-2 | Moving Picture Experts Group-2 | Recommended |
|  | MPEG-4 | Moving Picture Experts Group-4 | Recommended |
|  | MP3 | MPEG-1 Audio Layer 3 | Recommended |
|  | AAC | Advanced Audio Coding | Recommended |
| Moving picture stream, audio stream | (.asf), (.wma), (.wmv) | Microsoft Windows Media Player formats (.asf), (.wma), (.wmv) | Recommended |
|  | (.ra), (.rm), (.ram), (.rmm) | Real Audio/Real Video formats (.ra), (.rm), (.ram), (.rmm) | Recommended |
|  | (.avi), (.mov), (.qt) | Apple Quicktime formats (.avi), (.mov), (.qt) | Recommended |
| Animation | GIF v89a | Graphic Interchange (.gif) version 89a | Recommended |
|  | (.swf) | Macromedia Flash format (.swf) | Recommended |
|  | (.swf) | Macromedia Shockwave format (.swf) | Recommended |
|  | (.avi), (.qt), (.mov) | Apple Quicktime formats (.avi),(.qt),(.mov) | Recommended |
| Mobile content | WML v2.0 | Wireless Markup Language version 2.0 | Compulsory |
| Character set and encoding | ASCII | American Standard Code for Information Interchange | Compulsory |
| Vietnamese character set | TCVN 6909:2001 | TCVN 6909:2001 “Information Technology - 16-bit Coded Vietnamese Character Set” | Compulsory |
| Data compression | Zip | Zip (.zip) | Compulsory application of one or both standards |
|  | .gz v4.3 | GNU Zip (.gz) version 4.3 |  |
| Client-side scripting language | ECMA 262 | ECMAScript version 6 (6^th^ Edition) | Compulsory |
| Web content sharing | RSS v1.0 | RDF Site Summary version 1.0 | Compulsory application of one of the two standards |
|  | RSS v2.0 | Really Simple Syndication version 2.0 |  |
|  | ATOM v1.0 | ATOM version 1.0 | Recommended |
| Network Protocol Standard | JSR 168 | Java Specification Requests 168 (Portlet Specification) | Compulsory |
|  | JSR286 | Java Specification Requests 286 (Portlet Specification) | Recommended |
|  | WSRP v1.0 | Web Services for Remote Portlets version 1.0 | Compulsory |
|  | WSRP v2.0 | Web Services for Remote Portlets version 2.0 | Recommended |
| **Information security standards** | | | |
| Email security | S/MIME v3.2 | Secure Multi-purpose Internet Mail Extensions version 3.2 | Compulsory |
|  | OpenPGP | OpenPGP | Recommended |
| Transport layer security | SSH v2.0 | Secure Shell version 2.0 | Compulsory |
|  | TLS v1.2 | Transport Layer Security version 1.2 | Compulsory |
| File transfer security | HTTPS | Hypertext Transfer Protocol Secure | Compulsory |
|  | FTPS | File Transfer Protocol Secure | Recommended |
|  | SFTP | SSH File Transfer Protocol | Recommended |
| Mail transfer security | SMTPS | Simple Mail Transfer Protocol Secure | Compulsory |
| Message access service security | POP3S | Post Office Protocol version 3 Secure | Compulsory application of one or both standards |
|  | IMAPS | Internet Message Access Protocol Secure |  |
| DNS security | DNSSEC | Domain Name System Security Extenssions | Recommended |
| Network layer security | IPsec - IP ESP | Internet Protocol security với IP ESP | Compulsory |
| Wireless network security | WPA2 | Wi-fi Protected Access 2 | Compulsory |
| Encryption algorithm | TCVN 7816:2007 | Cryptographic technique - Cryptographic algorithms - Data Encryption Algorithm AES | Recommended |
|  | 3DES | Triple Data Encryption Standard | Recommended |
|  | PKCS #1 V2.2 | RSA Cryptography Standard - version 2.2 | Recommended, using RSAES-OAEP scheme for encryption |
|  | ECC | Elliptic Curve Cryptography | Recommended |
| Digital signature algorithm | PKCS #1 V2.2 | RSA Cryptography Standard - version 2.2 | Compulsory, using RSASSA-PSS  scheme for signature |
|  | ECDSA | Elliptic Curve Digital Signature Algorithm | Recommended |
| Digital signature hash algorithm | SHA-2 | Secure Hash Algorithms-2 | Recommended |
| Key transport algorithm | RSA-KEM | Rivest-Shamir-Adleman - KEM (Key Encapsulation Mechanism) Key Transport Algorithm | Compulsory |
|  | ECDHE | Elliptic Curve Diffie Hellman Ephemeral | Recommended |
| User authentication solution | SAML v2.0 | Security Assertion Markup Language version 2.0 | Recommended |
| XML message exchange security | XML Encryption Syntax and Processing | XML Encryption Syntax and Processing | Compulsory |
|  | XML Signature Syntax and Processing | XML Signature Syntax and Processing | Compulsory |
| XML public key management | XKMS v2.0 | XML Key Management Specification version 2.0 | Recommended |
| Personal information security protocol | P3P v1.1 | Platform for Privacy Preferences Project version 1.1 | Recommended |
| **Public key infrastructure** | | | |
| Cryptographic message syntax for signing and encrypting | PKCS#7 v1.5 (RFC 2315) | Cryptographic message syntax for file-based signing and encrypting version 1.5 | Recommended |
| Cryptographic token information syntax | PKCS#15 v1.1 | Cryptographic token information syntax version 1.1 |  |
| Private-key information syntax | PKCS#8 V1.2 (RFC 5958) | Private-Key Information Syntax Standard version 1.2 |  |
| Cryptographic token interface | PKCS#11 v2.20 | Cryptographic token interface standard version 2.20 |  |
| Personal information exchange syntax | PKCS#12 v1.1 | Personal Information Exchange Syntax version 1.1 |  |
| Certificate revocation list format | RFC 5280 | Certificate Revocation List Profile |  |
| Digital certificate format | RFC 5280 | Public Key Infrastructure Certificate |  |
| Certification request syntax | PKCS#10 v1.7 (RFC 2986) | Certification Request Syntax Specification version 1.7 |  |
| On-line Certificate status protocol | RFC 6960 | On-line Certificate status protocol |  |
| Time stamping protocol | RFC 3161 | Time stamping protocol |  |
| Time stamping services | ISO/EEC 18014-1:2008 | Information technology Security techniques - Time stamping services |  |
|  | ISO/EEC 18014-2:2009 | Part 1: Framework |  |
|  | ISO/EEC 18014-3:2009 | Part 2: Mechanisms producing independent tokens |  |
|  | ISO/EEC 18014-4:2015 | Part 3: Mechanisms producing linked tokens |  |
|  |  | Part 4: Traceability of time sources |  |
| Web services security | WS-Security v1.1.1 | Web Services Security: SOAP Message Security Version 1.1.1 | Recommended |
| Incident object description exchange format | RFC 7970 | The Incident Object Description Exchange Format version 2 (IODEF) | Recommended |
