# Supplementary material for: Digital Health Policy and Programs for Hospital Care in Vietnam: Scoping Review
Source: J Med Internet Res. 2022 Feb 9;24(2):e32392. doi: 10.2196/32392 (PMC8867296; doi:10.2196/32392)
Supplement: Multimedia Appendix 5 [file jmir_v24i2e32392_app5.doc]

## Multimedia Appendix 5

**Circular 48/2017/TT-BYT - Regulations on data exchange in management and reimbursement of health insurance claims**

There are two portals that organizations need to submit their data to, the health insurance portal from the VSS and the health data portal from the MoH. While the VSS’s portal serves the health insurance claiming and investigation purposes, data submitted to the MoH’s portal will be stored in the national database for research and policy making purposes. A healthcare facility will submit the same dataset to both portals following a set of guidance for data sharing and formatting.

Requirements for data format

The circular categorizes data used for health insurance claiming purpose into two groups, input and output data. Input data is the data collected and stored in the IT system while output data is compiled and exported from the IT system for sharing purposes. Specifications for these data are as followed.

| Data coding and nomenclatures | The terminology and service coding systems of the MoH is required for both input and output data |
| --- | --- |
| Data format | Data must be formatted in XML format and uses the UTF-8 encoding system |
|  | One XML file can contain one or multiple health packages containing the information of one visit bout, even when the client has multiple insurance IDs |

Data sharing protocols

The data can be shared to the portals through one of these protocols

- Protocol 1: via the web service.
- Protocol 2: data sync from the software installed on a workstation computer.
- Protocol 3: direct data import.
- Protocol 4: using the File Transfer Protocol (FTP).

In either of these protocols, healthcare facilities must ensure data integrity is maintained.

Instant data sharing and feedbacks

Healthcare facilities submit the data to the health insurance portal “instantly” after the end of a doctor’s visit, an outpatient treatment period, or an inpatient discharge. These data do not need to go through electronic verification.

Once received the data, the health insurance portal must reply the sender with a confirmation of receiving data along with other information that is:

- Information about the current status of the health insurance account.
- The patient’s medical history for the last six months, including:
  - Time points of the last visits.
  - The primary diagnoses and comorbidities classified with ICD-10 or the traditional medicine classification system.

Reviewed data sharing and feedbacks

A secondary data submission is carried out within seven working days after ending a healthcare activity or a treatment period. The data that have been transferred instantly will be reviewed, corrected for any discrepancies, and electronically verified. Data will then be submitted to two portals, the health insurance portal and the health data portal. If the data is generated in the end of a month/quarter/year, data submission should be conducted by the fifth day of the next month.

The portals must notify the sender if data is successfully received. The health insurance agency will assess the claim information and reply to the healthcare facilities in details about the investigation results within seven working days since data arrival. Conflicts and queries related to the claim, if any, must be clearly indicated in the relevant data field of the XML file to facilitate effective communication and timely justification between the healthcare facilities and the health insurance agency.

Delay in data sharing

Delay in data sharing is acceptable in some particular cases. Following is the policies related to sharing delay.

| Cases when delay in data sharing is acceptable | When there are unpredictable technical errors of the information systems that obscure data transfer and feedbacks |
| --- | --- |
|  | When there is an interruption of power supply or Internet connection, or incapable IT infrastructure that impedes data transfer and feedback |
| Notification of the interruption | Healthcare facilities must notify the portal admins about the issues, and vice versa via phones, emails or faxes |
|  | Data transfer and feedback must be continued after the issues have been resolved |
| Provide alternative communication solutions when IT infrastructure is incapable | The healthcare facility and the VSS must agree on the alternative solutions for data transfer and address them clearly in contract then inform the supervising organization about the substitution |

Data security and data management

The data exchange activities and the data itself are protected and managed in compliance with relevant cybersecurity regulations and healthcare regulations.

Organizations engaging in the data exchange activities must be proactive and coordinative, within permitted responsibilities, to ensure safety, security, accuracy, and integrity of the data.
