# Supplementary material for: Digital Health Policy and Programs for Hospital Care in Vietnam: Scoping Review
Source: J Med Internet Res. 2022 Feb 9;24(2):e32392. doi: 10.2196/32392 (PMC8867296; doi:10.2196/32392)
Supplement: Multimedia Appendix 6 [file jmir_v24i2e32392_app6.doc]

## Multimedia Appendix 6

**Decision 4159/QD-BYT year 2014 on Guidance on ensuring security of electronic health data in health organizations**

***Organization’s information security policies***

Organizations must have local information security policies for their IT system. The policies need to be approved by the organization’s management board and compliant with relevant regulations of the government. The policy’s structure should at least cover the following sections:

- Background (scope, applicable groups, concepts, aims).
- Detail policies (key points, standards, specific requirements).
- Roles and responsibilities of relevant stakeholders.
- Policy implementation.

The policies should be annually reviewed and modified if needed to ensure its appropriateness, adequacy and efficiency.

***Intranet and Internet security***

Organizations must apply measures to safeguard their intranet and Internet security, including:

- Solutions to detect and prevent intrusion and malwares.
- DoS prevention.
- Verification solutions for wireless network.
- Network partition measures to manage access and provide quick and efficient access.
- Backup for important components in the system.
- Demand evaluation to ensure suitable bandwidth for the local and Internet network.
- Regular updates for the network and security devices.
- Ensuring an adequate supply of trustworthy security and network infrastructure for the organization.

***Server and system software***

The requirements for server and system software are shown in the table below:

| Server infrastructure | The server and related equipment have good performance and efficiency for implementing IT activities. |
| --- | --- |
| Requirements for server | High availability and flexible back-up mechanism. |
|  | Having separate and suitable server room, strict protection and supervising of server room, suitable physical environment e.g. power supply and room temperature for uninterrupted operation, fire safety solutions, and assigning clear roles and authorization for personnel having access to the room. |
| Remote access, security, and working environment for the server | On-site or remote access to the server must be protected with passwords or other means of control; server should be assigned to a separate and proper network partition. |
| Intrusion and virus prevention | Have protocols to detect and prevent intrusion, malwares and virus for server |
| Requirements for system software | Operating system installed on server have legal license or are popular open-source OS such as UNIX, LINUX |
|  | System software have technical documents |
|  | Regular checks and updates of software patches |

***Workstation computers***

Workstation computers in the organizations need to be protected from a variety of threats including unauthorized access, viruses, confidentiality breach, and system errors:

- All workstation computers including desktops and laptops must be password-protected and/or secured with other measures.
- Databases or folders keeping important health information must be password-protected.
- Organizations must have protocols to detect and prevent workstation computers from intrusion, malwares, and viruses.
- Organizations must implement measures to protect data in workstation computers if they are connected to the Internet.
- Workstation computers at places where communication with customers happens need to be prevented from unwanted view of confidential information. Screen lock when not in use setting should be turned on.
- Frequent updates of error fixes and upgrades of the operating system.

***Application software***

Application software security set out responsibilities of the software provider, the supporting agency and the host organization in preserving cybersecurity related to application software.

| Security requirements from the manufacturer/provider | Security requirements from the manufacturer/provider need to be clearly addressed in the technical document. These requirements should be thoroughly adhered to during using the software |
| --- | --- |
| Risk assessment and prevention | Risks should be assessed, and risk prevention measures should be continuously conducted before the software is installed until it is in use |
| Security testing and assessment | Before accepting the software, relevant security testing and assessment must be conducted and documented. The test should take place in a separate environment not affecting the organization’s ongoing activities |
| Account management | Strict management of all the user accounts |
| Version management and updates | Evaluate efficiency, effect and risks when integrating with other software and when upgrading to a new version |
|  | New versions should experience security testing and earn authority’s approval before being implemented |
|  | Have recovery protocols if issues occur during software upgrade |
|  | The upgrade procedure should be documented with relevant purpose, version, time, person conducting the upgrade |
|  | Software versions should be well managed and stored in a secured place |
|  | The upgrade procedure should be supported with detailed guiding documents |
| Management of source program | Assign personnel to manage the software’s source program |
|  | Access to source program must be authorized and documented |
|  | Source program must be kept safely at two separate locations |
|  | The software provider and the supporting agency must provide assurance of malware-free to the customer |
| Software’s license | Encourage using licensed software, strictly avoid using illegal license. The organization or individuals must be responsible for consequences of using illegal software |

***Email***

Email security requires good email management from the organization as well as safe ownership and practice from the users:

- Using public email addresses to exchange work emails should be avoided while work email addresses should not be used for personal purposes.
- Regular password changing and using non-editable document formats should be encouraged.
- Emails should only be used by the authorized users. Passwords for emails should have good complexity and can only be changed by the authorized person.
- Staff’s emails should be locked or deleted when they stop working for the organization.
- The organization must have measures to ensure the email system’s security and readiness, as well as junk mail prevention measures in the intranet and Internet environment.

***Database***

- Organizations must only use database management systems (DBMS) with legal license and origin, or popular open-source DBMS such as MySQL, PostgreSQL, MongoDB.
- DBMS need to satisfy the following criteria: stability, being able to store and handle the organization’s data, having measures to protect and authorize access to the DBMS resources.
- Regular updates to fix DBMS’s errors.
- Organizations should develop backup and recovery protocols for the database.
- Organizations should implement user authorization with clear assignment of roles for individuals accessing the database. Logging activities on the database is encouraged but this should not interfere with data processing speed.
- Protocols to prevent intrusion and unauthorized access should be implemented.

***Backup and recovery***

The data backup and recovery requirements below cover workstation computers, servers, and software in an organization.

| Data on workstation computers | Important data should be backed up right after modification to allow for recovery if needed |
| --- | --- |
|  | Important data needs to be backed up before changes or upgrades in the operating system are made |
|  | Backup devices and recovery procedures should be checked regularly to ensure preparedness |
|  | Backup data must be stored in a safe location separated from the original data and unauthorized personnel. Important data should ultimately be stored separated from the host facility’s geographic location |
| Data on servers | Backup data and data recovery documents must be stored in a location separated from the installation location to avoid severe issues. Backup frequency, especially of important data, must be planned to meet the organization’s recovery needs |
|  | Backup devices should be regularly checked to ensure preparedness |
|  | Backup data should be stored at a location that has physical protection and meets the same protection standards applied for the main storage place |
|  | Storing duration for backups of important information and requirements for permanent backups should be identified |
|  | Database recovery should be regularly checked to ensure efficiency and capability to recover data within the time limit |
| Software | Original version of the software must be safely archived for quick reinstallation if needed |

***Data transmission***

Health facilities should use identification and encryption methods to protect their data during data exchange. Specific requirements in using these methods are as followed:

- Identification methods allowed by Vietnam law and the MoH should be used in data transmission.
- Using the encryption methods suitable for the security configuration and processing capacity of the information system.
- The encryption keys must be created, modified, distributed, and stored in a safe manner.
- Ensuring the encrypted data can be decrypted when needed.
- Developing policies about withdrawing, eliminating, and restoring encryption keys.

***User accounts***

This section addresses the good practice in user account management that organizations need to follow. Details are presented below.

| Access authorization policies | Developing organization’s policies for authorizing access right to network, server, application software, and database, covering from access requesting to access removal |
| --- | --- |
| Privileged account management | Developing policies in controlling and supervising access to the privileged accounts |
| Security requirements in user account management | Each user is only allowed to own one account |
|  | Defining access groups and authorizing accounts based on these groups |
|  | Passwords should be changed routinely (at least once a month) |
|  | The organization should have definitions for a strong password such as the limited character counts, required availability of both upper case and lower cases, or both alphabet and numeric characters |
|  | A temporary password is created for a new account and will be changed after first sign-in. If possible, no reuse of old passwords should be applied |
|  | Have procedures to instantly remove accounts and halt authorizations of staff who change their role or stop working for the organization |

***Remote access***

To secure remote access activities, organizations must make sure the following instructions are followed:

- User authentication and recognition capability of the security system:
  - Able to proactively identify the user and their access right.
  - At minimum, passwords are used to recognize users.
  - Important resources can be protected by more advanced solutions such as smart cards, token keys, and biometrics.
- Protection for data during transmission: encryption methods can be used to protect important data during transmission.
- Protection for resources in the network: implementing protocols for controlling resources subject to remote access.

***Elimination of storage devices***

Storage devices intended to be eliminated need to be handled appropriately to avoid unwanted data security issues.

- Before being eliminated, devices storing important health data need to be checked and ensured that any data or licensed software is removed or re-formatted.
- Storage devices no longer able to work but used to stored important data must be physically destroyed before elimination.

***Ensuring continuity of the IT system***

Uninterrupted operation of the IT system is an important objective that healthcare facilities need to achieve. Below is the recommended measures to maintain IT systems’ continuity:

- Developing and conducting plans to maintain continuity of the information system.
- Building data backup and recovery protocols.
- Ensuring data can be access quickly and uninterruptedly.
- Having network backup solutions.
- Having protocols to ensure continuity of the server system. Using technologies to support server availability is encouraged.

***Issue management***

Potential cybersecurity issues need to be prevented through appropriate protocols and techniques. Recommended measures are shown below:

- Organizations should develop internal IT issue management procedures. The procedures should be reviewed and updated with new issues and solutions at least every six months.
- Employing techniques to detect and solve DoS attacks such as firewall devices, intrusion detection and prevention devices, attack alert devices, and packet filter.
- Organizations should request the service providers to provide issue management protocols for their services.

***Human resource to ensure cybersecurity***

To ensure the system’s cybersecurity, organizations is required to the following guidance:

- The organization should assign at least one person with appropriate competency to the cybersecurity position.
- System administration, application software development and maintenance, and system operation tasks should be assigned to specific teams and individuals. No individuals should have the full authorization for the system except ones approved by the organization's head. Relevant responsibilities of these groups and individuals should be clearly addressed in the organization’s policy.
- Publicizing policies for strict management of system access on privileged accounts.
- Organizing trainings and continuous education for cybersecurity staff.
- Recruited staff should be educated about organization’s information safety policies.

***Supervising external IT providers and supporters***

Technicians from service providers or technical services who temporarily work with the organization’s IT system need to be supervised to avoid unwanted cybersecurity issues. In particular, organizations should develop and implement thorough protocols for supervising external technicians working with the IT system. These protocols must be approved by the organization’s authority.
