# Supplementary material for: Digital Health Policy and Programs for Hospital Care in Vietnam: Scoping Review
Source: J Med Internet Res. 2022 Feb 9;24(2):e32392. doi: 10.2196/32392 (PMC8867296; doi:10.2196/32392)
Supplement: Multimedia Appendix 7 [file jmir_v24i2e32392_app7.doc]

## Multimedia Appendix 7

**Laboratory Information Systems**

**Decision 3725/QD-BYT year 2017 on Guideline for implementation of laboratory information system in healthcare facilities**

***Information for new lab test orders***

LIS should be able to record the information of new orders by three groups, patient’s personal information, sample information, and order information. Data items for each group are presented below.

| Patient's personal information | Patient ID, administrative information such as name, age, sex, address, department, room, bed, and health insurance ID |
| --- | --- |
| Sample information | Sample code, type of sample, sample source, department ordering test, and sample receiving time |
| Test order information | Service code, service name, department and staff odering test; test order by individual test or by package of tests; capability to add test order to an existing sample |

***Data entry methods***

Data can be transferred to LIS via multiple methods such as:

- Laboratory staff manually enter data to LIS using the data entry interface.
- Information is sent from HIS to LIS.
- Information is read to LIS by scanning the codes (barcode, QR code, other types of code) printed on test referral sheets.

***Sample labelling***

LIS can create unique sample IDs that are used throughout the lab procedures. Information on sample labels must have sample IDs at minimum.

***Order status and history viewing***

LIS enables users to search, view, and print information related to a patient or a sample. The viewed information can be sample’s ID, patient name, sample status, time points, and test history.

LIS can support testing management tasks via managing lists of orders and related details and results. Progress of an order can be shown on LIS e.g. sample taken/not taken, sample processed/not processed, results available/not available.

***Queue management***

LIS has capability to manage queues of sample collection and queues of pending results. These queues can be displayed on screens to inform patients.

***Result management***

LIS’s result management capability involves the essential functions such as updating, archiving, displaying, summarizing, and printing test results as well as the extra functions e.g. alert, writing notes, and locking records. Details of these functions are as followed.

| Test and administrative information update, display, and archive functionality | Patient's administrative and clinical information |
| --- | --- |
|  | Information about the department and the doctor making the order |
|  | Information of the result: test name; date and time of ordering, collecting, and analyzing sample; test results by various indicators; time of announcing result; data entry person and other relevant information |
|  | LIS can integrate with lab machines to directly receive the results |
| Alert for abnormal results |  |
| Allowing users to write notes for the results (if necessary) |  |
| Locking results after completion |  |
| Managing and summarizing test results based on the standard templates in Decision 4069/2001/QD-BYT | Templates in Decision 4069/2001/QD-BYT: - Lab test voucher (27/BV-01) - Hematology result sheet (28/BV-01) - Template 29/BV-01 - Template 30/BV-01 - Template 31/BV-01 - Template 32/BV-01 - Template 33/BV-01 - Template 34/BV-01 - Template 35/BV-01 - Template 36/BV-01 - Template 37/BV-01 |
| Printing test results and export result documents to popular file formats | Some popular digital formats such as .doc, .docx, .xls, .xlsx, .pdf |

***Inventory management***

This functionality helps staff to manage and monitor the laboratory chemicals and supplies, including:

- Inventory management: reporting the inventory, generating warehouse input/output certifications, and inventory viewing.
- Keeping track of the supplies and chemicals taken from the inventory to run a test.
- Setting consumable standards of supplies and chemicals for each type of test.
- Summarizing the amount of supplies and chemicals used for each type of test.
- Summarizing the amount of supplies and chemicals used in each test device.
- Managing, summarizing and printing inventory reports.
- Summarizing supplies and chemicals by patch number, expiring dates. LIS can prioritize supplies and chemicals with the nearest expire dates.

***Interoperability between LIS and lab machines***

- LIS is able to integrate with lab machines to send orders and receive results automatically. In particular, there are two types of communication between LIS and lab machines:
  - In one-way communication, LIS can automatically receive results from the lab machines.
  - In two-way communication, LIS is not only able to automatically receive results from the lab machines, but also able to send orders and commands to the lab machines.
- LIS can define and automate the mapping process for lab test names for standardization.
- LIS can receive and read graphs from some lab machines such as hematology analyzers, electrophoresis equipment, and electrocardiograph machines, or can generate graphs from the data extracted from these machines.
- LIS can connect with the devices that can connect with popular lab machines in Vietnam via the standards such as RS232, RJ11, RJ45, USB.
- LIS can receive and store the images captured from the devices connected to it.

***Interoperability between LIS and HIS***

- LIS receives patient information and order information from HIS instead of manually entering these information to LIS; results are transferred from LIS to HIS
- LIS supports sending test orders to lab machines having two-way connection.
- LIS can exchange data with other laboratories and with other information systems to support management and administration purposes.

***Reporting and statistics***

Organizations can use LIS to produce summaries and reports to their managers, the MoH, or the VSS. Following is all types of reports that LIS should be able to generate.

| Generating summaries from specific lists | List of received samples |
| --- | --- |
|  | List of samples to collect |
|  | List of pending tests |
|  | Summaries for all tests |
|  | Chemical quotas |
| Managing and summarizing data, and exporting reports based on the registry formats in Decision 4069/2001/QD-BYT | The relevant registry formats in Decision 4069/2001/QD-BYT are:   - The lab registry (14/BV-01) - The peripheral blood cell test registry (15/BV-01) - The microbiology registry (18/BV-01) - The test result announcement form (20/BV-01) |
| Generating summaries and reports customized to the host organization and the VSS’ requests |  |

***Implementing terminology and service coding systems***

The MoH requires healthcare facilities to widely adopt the MoH terminology and service coding system when implementing LIS to create consistency between technical procedures, administration, and reporting. Below is the terminology and service coding lists that LIS are required to adopt:

- Lists of lab test indicators, lists of lab test indicator groups; lists of lab service categories; lists of lab techniques, lists of technical service categories; lists of lab sample categories; lists of lab instrument; lists of medical supplies, lists of blood and blood products (published by the MoH).
- Lists of hematology-transfusion, biochemistry and microbiology techniques in Circular 43/2013/TT-BYT and its amendments in Circular 21/2017/TT-BYT.
- LOINC standard (recommended in Decision 2035/QD-BYT year 2013).

In addition, LIS can be updated with extra terminology systems or newer versions of the installed systems.

***System administration***

The system administration functionality group includes the following functions:

| User administration | LIS user administration |
| --- | --- |
|  | Authorization |
|  | Audit management |
| Setting alert threshold for abnormal results |  |
| LIS configuration | Setting configurations for database connection |
|  | Setting configurations for work modes |
|  | Backup |
|  | Audit mode |
|  | Log in, log out and other related functions |

***IT infrastructure and human resource to operate LIS***

IT infrastructure

IT infrastructure conditions to implement LIS need to satisfy the relevant criteria addressed in Article 3 of Circular 53/2014/TT-BYT (Required conditions for provision of health IT activities). In particular, capacity of the network, server, and auxiliary devices to operate LIS must be ensured as followed:

- The local network is properly designed and implemented, with proper bandwidth.
- The server system should have appropriate performance ability and efficiency to operate the lab system. High availability and flexible backup mechanism is also required for the server system to operate LIS without interruptions.
- Auxiliary devices such as workstation computers and printers should be installed with suitable quantity and capability to cooperate with LIS and other systems.

Cybersecurity

Measures to protect cybersecurity during implementing LIS should seek to meet the requirements in Article 4 of Circular 53/2014/TT-BYT (Required conditions for provision of health IT activities) and Decision 4159/QD-BYT year 2014 (Guidance on ensuring security of electronic health data in health organizations).

IT workforce

At minimum, a facility must have one IT specialized staff with an associate degree or higher to administer and operate LIS.
