# Supplementary material for: Digital Health Policy and Programs for Hospital Care in Vietnam: Scoping Review
Source: J Med Internet Res. 2022 Feb 9;24(2):e32392. doi: 10.2196/32392 (PMC8867296; doi:10.2196/32392)
Supplement: Multimedia Appendix 8 [file jmir_v24i2e32392_app8.doc]

## Multimedia Appendix 8

**Hospital Management System**

**Decision 5573/QD-BYT year 2006 on Requirements and functional modules for hospital management software**

General requirements

HMS development and implementation must be based on the current laws and regulations of the government and the MoH. The specific requirements are given as followed:

- HMS development and implementation must comply with the current laws from the government.
- The HMS must satisfy all the hospital administrative tasks regulated by the MoH.
- Data and template configuration used in HMS must ensure appropriateness with data structure of the reporting and medical record system of the MoH.
- The MoH Terminology and Service Coding System should be universally used in the facility to maintain consistency throughout healthcare delivery, reporting and payment. Double data entry should be avoided.
- Following MoH requirements and standards in reporting and managing medical records.

Technical requirements

- The HMS can communicate with the Medisoft 2003 software, or can export data and generate reports that satisfy Medisoft 2003’s report standards.
- The HMS can communicate with the Health Insurance software, or can export data and generate reports requested by the Vietnam Social Security.
- The facility must have solutions to maintain security of HMS including ensuring data safety and information security, having user authorization and authentication, and capability to strictly control user activities and prevent unauthorized access. The security system must have at least 3 layers: system, database, and user.
- Using UTF-8 for character encoding.
- Requirements for operation system, database and software development language:
  - The development of HMS that can run on free operation systems and database management systems are encouraged.
  - HMS has data backup and recovery functionality.
  - Having solutions for the database management issue.
  - Legal license of the development language can be proved.
  - Maintaining objectivity and honesty between database and reports.
- The software's design is openness-oriented and easy for troubleshooting, maintenance and upgrading.
- Advanced technologies and techniques are encouraged in developing and implementing HMS.
- Some nomenclature systems and health IT standards recommended for HMS are listed below:

| **Nomenclature systems** | |
| --- | --- |
| **System** | **Author organization** |
| The List of Administrative Units of Vietnam | Vietnam Government |
| The Hospital IDs List | MoH |
| The First-line Healthcare Facility IDs for Health Insurance Users | VSS |
| The Anatomical Therapeutic Chemical Classification System (ATC) | WHO |
| WHONET | WHO |
| ICD-10 | WHO |
| **Health IT standards** | |
| **System** | **Author organization** |
| HL7 messaging* | HL7 |
| Digital Imaging and Communications in Medicine (DICOM) |  |
| Picture Archiving and Communications Systems (PACS) |  |

*If HL7 standards are currently not supported in the HMS, the provider must commit on providing technical documents or supports to help the hospital's system connect with other information systems of the hospital, or the Department of Health and the MoH systems.

HMS functional modules

To adequately address the administrative tasks in a hospital, an HMS need to be incorporated with a wide range of functionalities. This guideline presents eight modules that an HMS should have, of which the constituent functionalities are explained as followed.

***Outpatient management module***

The purpose of this module is to manage all administrative and health service data of the patients. These data can be accessed in the whole hospital, and is used at the next patient’s visits. Key functionalities and instructions are provided below:

| Registration management | Generate and distribute unique patient IDs which will be used in the next time the patient visits |
| --- | --- |
| Manage the data recorded by the MoH standard medical record template | Demographics data: fullname, date of birth/age, 4 levels of address: village/house number - commune/ward/street - district - province/city |
|  | Patient group data: cost-free, pay, health insurance, poor group, children under 6 years old, and other groups |
|  | Health insurance related data (regulated by the VSS): health insurance ID, first-line healthcare facility, valid date, beneficiary code, first health insurance provider, reasons of care seeking |
|  | Referral place info: ID, facility's name etc. |
| Room management | Diagnosis management by ICD-10 (4 characters): medical history, previous diagnosis, primary diagnosis, other diagnosis |
|  | Doctor visit management: date & time of examination, doctor's name, data entry staff's name |
|  | Order management (lab tests, medical imaging, treatment) |
|  | Prescription management, including archiving and printing |
|  | Manage doctor's decisions: outpatient treatment, admission, clinic referrals |
|  | Print the MoH examination sheet for patients admitted to hospitals |
| Outpatient management | Outpatient medical record management |
|  | Outpatient order and service management |
|  | Statistics for outpatient duration |
| Manage patients monitored at the outpatient ward | Medical professional management |
|  | Service management |
| Outpatient test management | |
| Pharmacy management | |

***Inpatient management module***

This module manages data of the patients admitted to the inpatient departments. Data collected includes administrative data, diagnoses, room management, and surgery management. Details of each functionality are as followed.

| Patient information management | All administrative information in the admission sheet and the medical record template from the MoH. |
| --- | --- |
| Disease information management | Adopt the ICD-10 with 4-digits coding. |
|  | Diagnosis management: previous diagnosis, outpatient diagnosis, primary diagnosis, accompanied diagnosis, medical history, main ward admission diagnosis, main ward referral diagnosis, main diagnosis at discharge, diagnosis at death, and diagnosis at autopsy. |
| Ward and room management | Bed management: number of beds, bed types, fees, tracking occupation of each bed type. |
|  | Ward discharge and referral to another ward. |
|  | Hospital discharge and referral to another hospital. |
| Surgery management | Organize surgery schedule: patient name, surgery time, main surgeon, main anaesthetist, and other surgery attendants; surgery types and cost. |
|  | Manage information recorded in the surgery info sheet and surgery outcomes. |
| Reporting and statistics | Summarize and export treatment reports by the 11 hospital reporting templates. |
|  | Generate reports under requests from the VSS and other agencies (if necessary). |
|  | Generate reports under requests from the host hospital. |

***Medical test management module***

It is noted that this module can be implemented by phases that reflect the hospital’s resource and information infrastructure. Priority should be given to order management (for summary and cost calculation), followed by result management (for clinical practice and electronic health records) and communicating with lab machines. This is considered a complex module that requires integration with other modules such as outpatient management, inpatient management, pharmacy and inventory management, and financial management. More guidance is presented below.

| Use the terminology and coding system from the MoH to manage medical tests | |
| --- | --- |
| Outpatient test management | Manage test orders from the outpatient ward, including patient ID, patient name, name of clinic making order, name of test, order time, name of doctor making order |
| Inpatient test management | Manage orders by patient |
|  | Manage results by patient |
|  | Complications occurred during a procedure |
|  | Manage and exchange data collected from the test machines (if available), such as images, videos, audio |
| Result management at the lab test and medical imaging departments | Manage and store all the results available at the department, together with the patient information |
|  | Manage administrative and clinical information |
|  | Manage information related to the ward and personnel making orders |
|  | Result information: name of test, ordering time, sampling time, sample processing time, name of the person conducting the test, test result, result announcement time, name of data entry staff etc. |
|  | Interoperability with test machines for direct result receipt should be aimed at. |
| Test cost management | |
| Supply and chemicals management | |
| Reporting and data export | Reports for testing activities can be generated under hospital's regulated templates. |
|  | Can generate reports requested by the hospital |

***Pharmacy management module***

The pharmacy management module aims to provide effective and safe management of the drug inventory in the hospital. Following is the key functionalities that this module entails.

| Drug and supply management | Have a standard terminology system for drugs and supplies applicable for the whole hospital. |
| --- | --- |
|  | Manage drug expired dates, utilizing a tool to monitor the expired dates and alert to drug expiration. |
|  | Able to withdraw drugs under the requests from the Pharmacy Administration Agency. |
|  | Have a pharmacopoeia to guide drug prescribing. |
| Inventory management | Develop catalogs to manage drug inventory import and export. Build a platform to create, edit, and remove the catalogs. |
|  | Have a data entry UI that can track inventory input by sources such as from providers, returned from the wards, produced internally, and record related information required by the MoH. |
|  | Have a data entry UI that can track types of inventory output such as return to providers and small-amount export, and record related information required by the MoH. |
|  | Have a data entry functionality to add products used for other output purposes such as disease prevention, scrapping, and liquidation. For each of these cases, design a suitable function to verify output, including generating output certificates and output vouchers. |
| Drug delivery management | Have a platform to manage drug delivery based on prescriptions, in which patients with health insurance and outpatient patients can be distinguished. In some cases, copies of outpatient prescriptions can be created. |
|  | The module can anticipate the needed drugs based on electronic data of medical records. Can differentiate between reserved by treatment demands and drugs reserved by default. |
|  | Have a functionality to return drugs and make related verification processes when the patient dies, changes drugs or withdraws from treatment. |

The guideline also set specific requirements for the module as well as the inventory management practice:

- The import and export process must adhere to the first in first out method, and be based on drug expiration dates as well as other regulations on keeping and delivering drugs.
- The module is able to manage inventory input-output and drug delivery by financial sources such as public funding, health insurance funding, and charity funding.
- The module has capability to generate quick and accurate summaries and reports of import, export and balance of inventory.
- The module has search functionality based on various criteria.
- The printable templates and registries in the module must adhere to the relevant pharmacy regulations.
- The module is able to generate reports of hospital pharmacy activities under the formats required by the MoH, and other reports requested from the Department of Health and the hospital.

***Hospital fee and health insurance management module***

This module is considered a crucial component of a HMS. It communicates with other HMS's modules, and is installed at the finance department and payment points in the hospital. Hospitals employing HMS should not use other software to manage hospital fees and health insurance to avoid double data entry and waste of resources. Detailed guidelines for the module’s functionalities are provided below.

| Adopt a unique service terminology system in management | Use the terminology and service coding system from the MoH. |
| --- | --- |
|  | Manage prices of treatment services based on current regulations from the MoH and the VSS. |
| Publicize costs for patients | Able to calculate hospital costs for any patient, at any point of time, under any payment protocol. |
| Manage costs and payments by specific groups | Direct payment group: self-pay patients, poor patients, autonomic finance management. |
|  | Indirect payment group: health insurance-covered groups (whole-cover and partial-cover), under-6 children. |
|  | Free group. |
|  | Other groups. |
| Outpatient cost management | Examination fee, medical test fee, surgery fee, treatment fee. |
| Inpatient cost management | Management of advance fee (for direct payment group). |
|  | Management of treatment costs, including medication, blood and fluid transfusion; bed fee; surgery; medical tests. |
|  | Publicize everyday costs: able to calculate patients' treatment costs anytime. |
|  | Able to print the standardized and customized invoices. |
|  | In health insurance reimbursement management, the module is able to distinguish between costs covered and not covered by health insurance. |
| Hospital cost management for patients with health insurance | Able to distinguish between costs covered and not covered by health insurance. |
|  | The software can generate patient’s datasets that meet the technical criteria from the VSS and can print related reports to serve health insurance reimbursement purpose. |
| Printing invoices and financial reports | Able to print invoices customized to the hospital's characteristics. |
|  | Able to print various types hospital cost reports (for custom service), including examination fee report, advance fee report, total cost report. |
|  | Able to summarize and print financial reports of hospital costs and health insurance. |

***Human resource and salary management module***

This module features three main functionalities as listed below.

| Human resource management | Manage staff's personal information |
| --- | --- |
|  | Manage training and education programs |
|  | Manage contracts |
|  | Able to search details of each staff bio (career, education, family background, abroad trips) |
|  | Statistics and reporting: Generate and print human resource reports under standardized formats from the MoH and customized reports requested from the hospital. For instance, demographics summaries, salary summaries. |
| Salary and social insurance management | Manage salary scales and incentives of each staff |
|  | Social insurance management |
|  | Timekeeping system: by month, overtime, surgery, shifts |
| Statistics and reporting | Manage and print reports requested from the state finance agency such as salary reports and timekeeping reports |
|  | Common templates and reports for human resource and salary management system |

***Supervision module***

This module is to manage the supervised facilities. Main functionalities include:

- Managing training and education programs of supervised facilities.
- Managing primary care and routine health examination activities from lower-level facilities (if available).
- Managing health programs (if applicable).
- Managing supervision reports.

***Medical device management module***

The main functionalities of this module is given as followed.

| Manage medical devices with a standardized nomenclature system | Build a standardized medical device nomenclature system that is used for the whole hospital. |
| --- | --- |
|  | Have an inventory management functionality for medical devices. |
|  | Have a functionality that allows creating, editing, and removing items in the standardized list. |
| Managing medical device usage status | Able to manage current usage status of the medical devices and generate related reports. |
| Managing new devices | Have a data entry functionality for receipt of new devices with specifications for devices received from the providers and devices received from the hospital's wards. |
| Managing device distribution | Have functionality to manage device distribution in the hospital, and device movement between the wards and to other hospitals. |
| Managing device maintenance | Have functionality to manage device fixing and maintenance. |
| Managing device upgrade | Able to identify the technical upgrades that increase device's value. |
| Managing device liquidation | Have liquidation management functionality. |
| Module upgrade capability | Able to add functionality items to meet the latest regulations from the MoH and the DoH. |
| Calculation and searching | Able to automate some calculation tasks and provide advanced searching functions based to multiple criteria. |
| Reporting | Able to generate and print reports under the standardized templates from the MoH. |
|  | Able to create reports customized to DoH's and hospital's requests. |
